# An updated review of experimental rodent models of pulmonary hypertension and left heart disease

**DOI:** 10.3389/fphar.2023.1308095

**Published:** 2024-01-08

**Authors:** Magdalena Jasińska-Stroschein

**Affiliations:** Department of Biopharmacy, Medical University of Lodz, Lodz, Poland

**Keywords:** animal models, left ventricular failure, pulmonary hypertension due to left heart disease, meta-research, rodents

## Abstract

Left heart disease (LHD) is the leading cause of pulmonary hypertension (PH). Its recent growth has not been matched by the design of therapeutic agents directly targeting the disease. Effective therapies approved for pulmonary arterial hypertension (PAH) have been shown to be inefficient in patients with PH-LHD. Hence, there is a need for an animal model that would closely mimic PH-LHD in preclinical experiments. The current study describes and compares a number of rodent models of left ventricular failure and their potential to induce PH. It also evaluates whether, and to what extent, common PH models could develop LV failure. Articles were identified in the Pubmed/Medline and Web of Science online electronic databases following the PRISMA Protocol between 1992 and 2022. Quality assessment was carried out using the SYRCLE risk-of-bias tool for animal studies. Publication bias across studies using Egger’s regression test statistic, was performed together with sensitivity analysis. A wide spectrum of protocols–135 studies and 207 interventions, was examined, including systemic hypertensive models, pressure-overload-induced HF, model of ischemic heart failure, and metabolic approaches based on high fat diet or metabolic syndrome. The most pronounced alterations in PH-related parameters were demonstrated for the common PH models, but were also seen in animals with LV failure induced by ischemic conditions, pressure overload or metabolic conditions. Models based on aortic banding, transverse aortic constriction (TAC), or with myocardial infarction (MI) caused by coronary artery ligation, demonstrated more pronounced worsening in PH due to LV failure; however, they also demonstrated poor survival, especially the ischemic-HF model. Common PH models, excluding prolonged exposure to monocrotaline, do not promote LV hypertrophy. Prolonged exposure to a high-fat diet, or a two-hit model of an obese ZSF1 rat combined with SU5416-induced pulmonary endothelial impairment (a VEGF receptor antagonist) worsened PH and impaired diastolic dysfunction. Due to the limited number of protocols, further trials are needed to confirm the utility of such approaches for modeling PH in subjects with metabolic syndrome. This would provide a clearer insight into the complexity of LHD, PH and metabolic disorders in PH-LHD, and thus accelerate the development of new therapies in clinical trials.

## 1 Introduction

Left heart disease (LHD) is the leading cause of pulmonary hypertension (PH) (Hoeper et al., 2016). In 2013, 61.7 million cases of HF worldwide were reported; in line with these findings, observational studies suggest an estimated prevalence of PH of 40%–72% in patients with reduced ejection fraction (HFrEF) and 36%–83% in those with preserved ejection fraction (HFpEF) (Guazzi and Naeije, 2017). Patients categorized as Group 2 PH, i.e., PH associated with left heart disease (PH-LHD), are more likely to manifest heart failure (HF) with HFpEF or HFrEF, valvular heart disease or post-capillary PH (Humbert et al., 2023).

PH-LHD patients are typically characterized by age above 70 years and at least two of the following: obesity, hypertension, dyslipidemia, and glucose intolerance/diabetes, as well as previous cardiac intervention or atrial fibrillation. LHD can cause chronic elevation of filling pressures in the left ventricular (LV) and backward pressure to pulmonary arteries, followed by vascular remodeling and increased pulmonary arterial pressures (PAP), pulmonary vascular resistance (PVR), transpulmonary pressure gradients, and secondary right ventricular (RV) hypertrophy (Delgado et al., 2005). Of note, in infants and children, congenital pulmonary vein stenosis has also been classified as PH group 2; this is a progressive disease which can eventually lead to PH and RV failure. In bilateral cases, the pressure load can cause significant morbidity and mortality (Humbert et al., 2023).

The increasing prevalence of PH patients with LHD in last few decades is unfortunately accompanied by a lack of therapeutic agents directly targeting the disease. Therefore, the primary strategy in managing PH-LHD is to optimize treatment of the underlying cardiac disease (Humbert et al., 2023). There is an increasing need to address the pathophysiology of PH-LHD, the complexity of the disease, its progression and potential therapeutic options. Animal models allow examination of physiological effects of cardiac function, and are useful in the development of therapeutic approaches. Hence, such models that closely mimic human disease, ideally those based on rodents due to their convenience and inexpensiveness, are often used in preclinical experiments in PH-LHD.

Recent literature provides a solid “qualitative” discussion on animal modelling in PH-LHD (Breitling et al., 2015; Liu and Yan, 2022). The authors summarize the characteristics of each model and discuss the advantages and limitations of individual approaches. They highlight a significant need for improvements to develop a more ideal model for the disease. This raises the question of whether there should be universal guidelines for the use of preclinical animal studies on PH-LHD. Such guidelines would allow researchers to select an appropriate, and reproducible, model depending on the specific research question being addressed.

The present review describes and compares the efficacy of the most popular approaches addressing PH-LHD from a quantitative point of view. The analyses are based on the results of a relatively large number of experimental protocols addressing the phenotypical characteristics of both PH and LV performance, and are supported by a variety of hemodynamic, echocardiographic, and histopathologic parameters. Review includes models featuring pulmonary hypertension from pressure-overload left ventricular failure (LVF), ischemic HF or metabolic diseases, as well as common PH models. It analyses in detail whether a) particular rodent models of heart failure can promote pulmonary hypertension and b) to what extent, and c) identifies the main determinants of severity in particular PH-LHD models (*primary end-point*). Additional analyses evaluate the risk of LV failure due to common PH inducers, with a special emphasis on monocrotaline, a widely-used compound (*secondary end-point*). The analysis also takes into account the commonly-reported parameters used in these models, such as animal age, body weight (BW) gain/loss, alterations in systolic blood pressure (SBP), blood glycemia, as well as covariates such as experimental period. The results obtained are expected to help add rigor to preclinical studies and improve their quality.

## 2 Material and methods

In brief, a literature search was conducted by two reviewers working independently. The articles were identified in the Pubmed/Medline and Web of Science online electronic databases following the PICO acronym and PRISMA protocol (1992–2022). The search included rodents (*population*). A variety of models of pulmonary hypertension (PH) displayed impairments in RV hemodynamics and structure that were expected to be accompanied with (or induced by) “left-heart disease” (LHD) (*intervention*). All studies included into analysis must have reported alterations in at least one PH-linked parameter and at least one parameter featuring LV heart failure (Vehicle) (*outcome*); such changes were also compared with another cohort—healthy animals (Sham, placebo control) (*comparator*).

More detailed information is provided in Supplementary Material.

### 2.1 Statistical analysis

#### 2.1.1 Primary end-point

For each pairwise comparison between healthy animals (Sham) and subjects intended to develop PH-LHD (Vehicle), the effect size was calculated with a 95% confidence interval (95% CI) as

$$D\left(diff\;in\;means\right)=\left(X_{PH-LHD}\right)\;–\;\left(X_{healthy}\right),$$ (1)

or

$$R\left(risk\;ratio\right)=\left(X_{PH-LHD}\right)\;/\;\left(X_{healthy}\right),$$ (2)

where X—mean response.

#### 2.1.2 Secondary end-point

For each pairwise comparison between healthy animals (Sham) and subjects suspected to develop left ventricular failure (LVF) due to common PH inducer (Vehicle), the effect size was calculated with a 95% confidence interval (95% CI) as

$$D\left(diff\;in\;means\right)=\left(X_{LVF}\right)\;–\;\left(X_{healthy}\right),$$ (3)

or

$$R\left(risk\;ratio\right)=\left(X_{LVF}\right)\;/\;\left(X_{healthy}\right),$$ (4)

where X—mean response.

The influence of particular methodological items (e.g., method to introduce PH-LHD, experimental period) on the final outcome was assessed by performing subgroup analyses and meta-regression. The experimental variability was compensated by a random-effects model. A Cochran’s Q statistics was used to assess the heterogeneity between studies. A *p*-value <0.05 was considered statistically significant. The analyses were conducted using STATISTICA 13.1 software.

#### 2.1.3 Survival analysis

Animal survival was assessed using the Kaplan-Meier method; the significance of any difference in mortality was verified according to an established protocol using the log-rank test.

### 2.2 Risk of bias, publication bias and sensitivity analysis

The evaluation of an individual study quality was performed using Systematic Review Centre for Laboratory Animal Experimentation—SYRCLE’s risk of bias tool for animal studies (Hooijmans et al., 2014).

Potential publication bias was assessed by Egger’s weighted regression, and Duval and Tweedie—“trim and fill” procedure.

In order to evaluate the influence of each study on the overall effect size, sensitivity analysis was conducted using the leave-one-out method, i.e., removing one study each time and repeating the analysis.

## 3 Results

**FIGURE 1:**
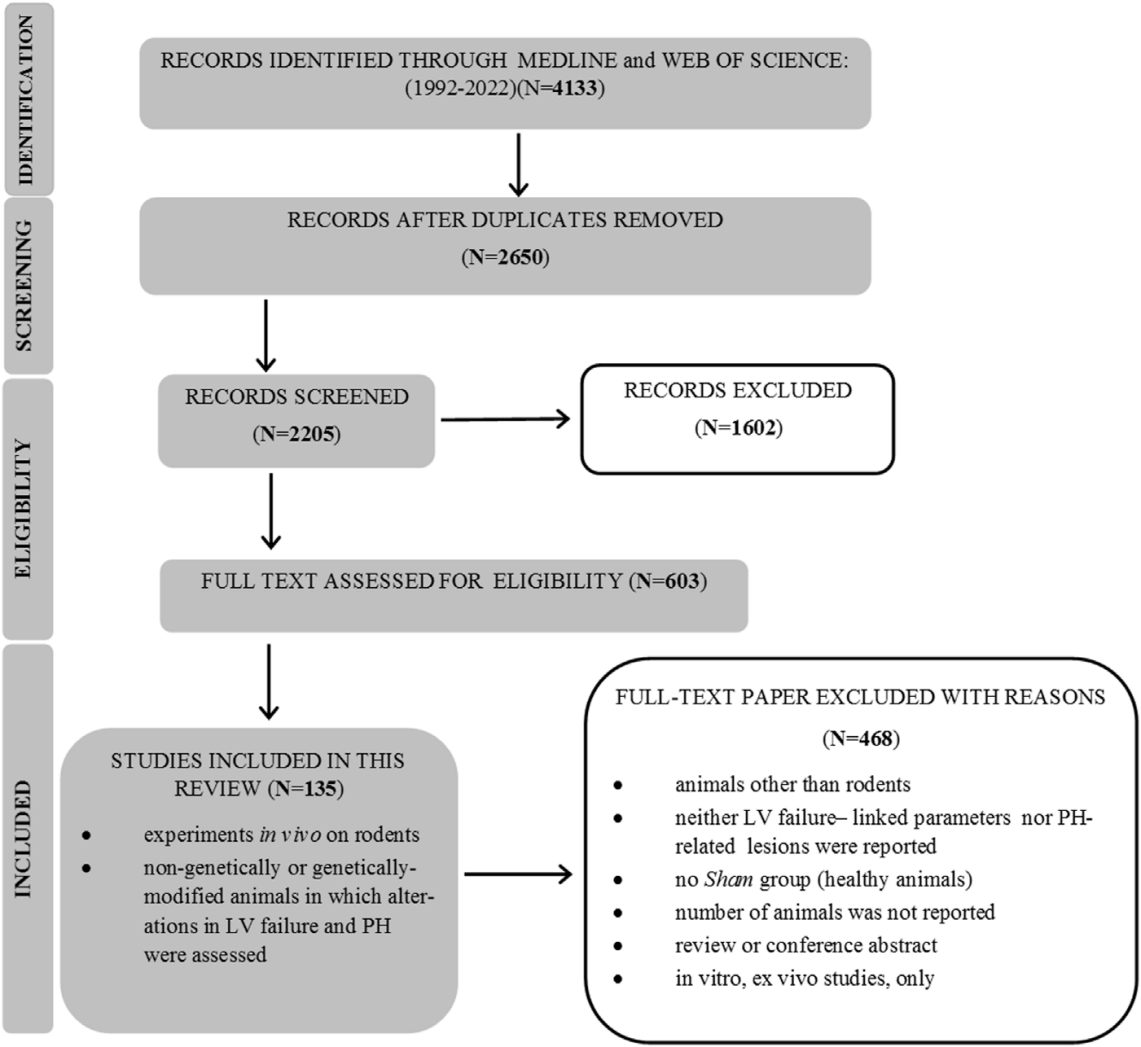
PRISMA flow diagram of the search study.

**FIGURE 2:**
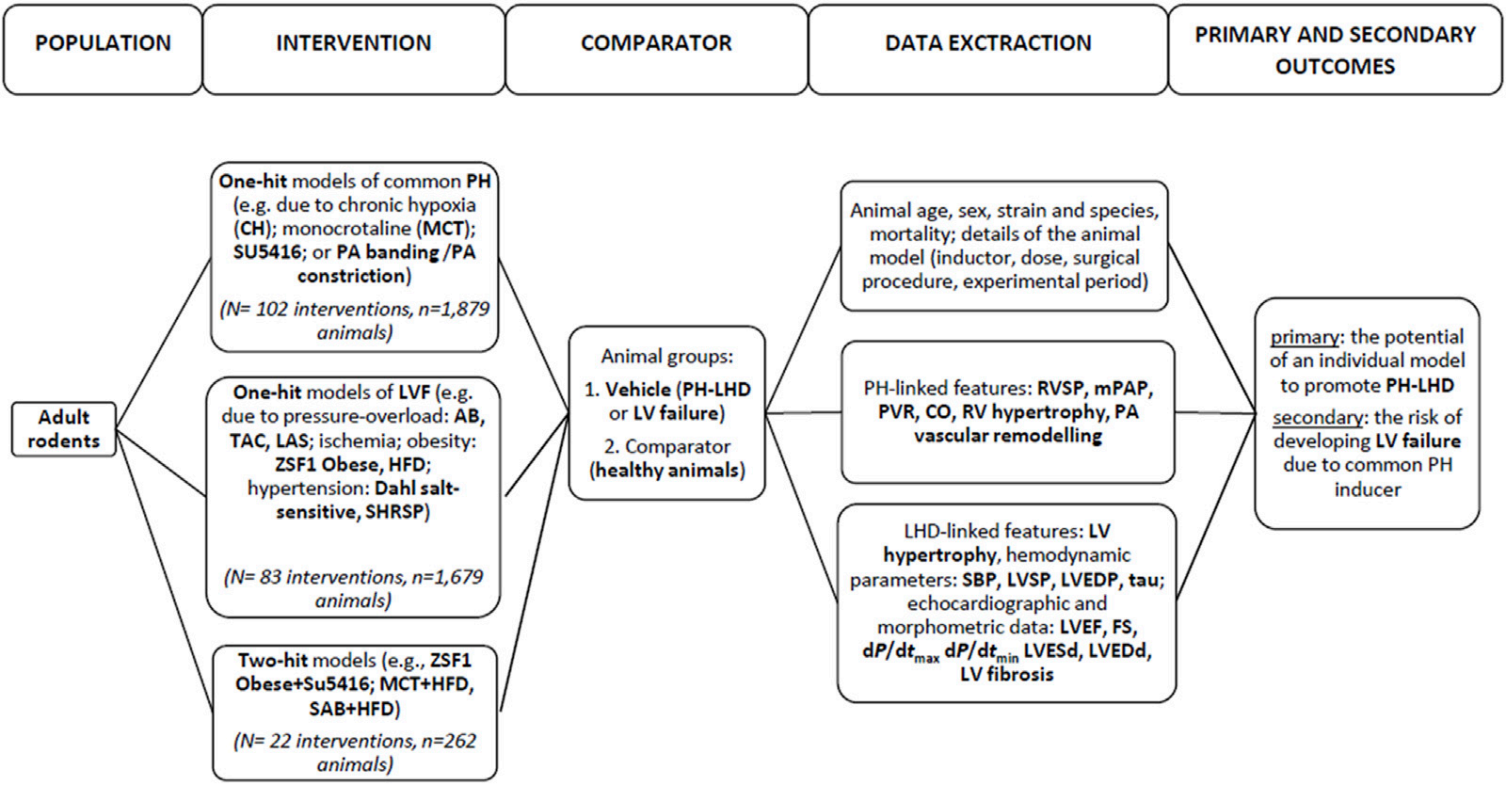
The study flow.

After an initial search, 4,133 papers were found, and 603 full-text publications were reviewed for the eligibility. Finally, 135 articles were included in this review, as demonstrated in Figure 1. Supplementary Material (Study references) includes the complete list of articles that were found to be relevant to this review. The study flow with regard to the animal model, parameters that feature PH-LHD, and the main study outcomes are presented in Figure 2.

### 3.1 Quality assessment of included studies

**FIGURE 3:**
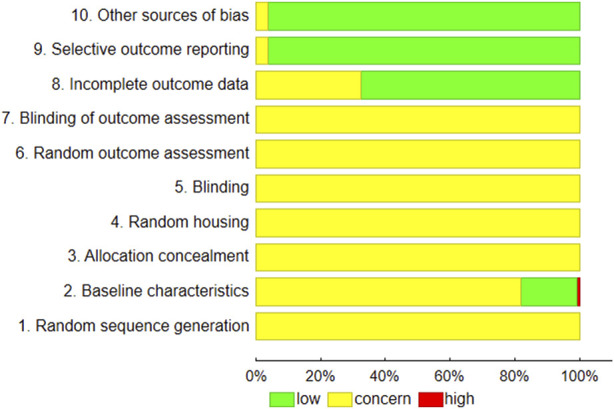
Summary of risk of bias according to the SYRCLE RoB strategy (Hooijmans et al., 2014).

Figure 3 demonstrates the results of the evaluation of risk of bias. The data for animal survival were provided in 15.8% of the papers.

The assessments of publication bias concerned several subgroups of animals receiving different treatments: exposure to chronic hypoxia (with SU5416), high-fat diets, monocrotaline injection, aortic banding, ligation of left coronary artery pulmonary artery or transverse aortic constriction. In addition, some studies were based on Dahl salt-sensitive and ZSF1 Obese rats. All examined a range of PH-LHD-related parameters. In most cases, the low possibility of missing studies, according to the trim and fill procedure, and a non-significant Egger test result (*p* > 0.05), indicates the absence of publication bias across the studies (Supplementary Table S1). The effect size was calculated according to Eqs 1–4.

The results of the sensitivity analysis are presented in Supplementary Table S2. The effect size was calculated according to Eqs 1–4. The following PH-linked parameters were considered: right ventricular systolic pressure (RVSP), mean pulmonary arterial hypertension (mPAP), right ventricular (RV) hypertrophy, pulmonary artery (PA) vascular remodelling. A number of outcomes related to left heart failure were also included in the analysis: LV hypertrophy (LVH), left ventricular systolic pressure (LVSP), left ventricular end-diastolic pressure (LVEDP), LV ejection fraction (EF), maximal rate of pressure rise (d*P*/d*t*_max_) and pressure fall (d*P*/d*t*_min_), left ventricular end-systolic diameter (LVESd), left ventricular end-diastolic diameter (LVEDd), as well as percentage of LV fibrosis. Most parameters were significantly increased by the interventions used to introduce PH-LHD. This effect was robust and statistical significance was not influenced by any single study included in the meta-analysis according to the parameters listed above, with an exception of the left ventricular end-systolic diameter, where the analyses were repeated after removing 1 out of 19 studies.

### 3.2 General design

In total, 135 studies and 207 interventions were identified, where the term intervention pertained to a separate comparison between Sham (healthy animals) and Vehicle related with a particular study protocol (animal species, model of PH-LHD, experimental period, etc.). They were performed on a total number of 3,820 animals. The most numerous models of PH with additional assessments of LV failure included monocrotaline injection (N = 68/207 interventions; 32.8%), animals with ligation of coronary artery (N = 24; 11.6%), aortic banding (N = 15; 7.2%), transverse aortic constriction (N = 12; 5.8%), exposed to high fat diet (N = 15; 7.2%), kept under chronic hypoxia conditions (N = 12; 5.8%) or with pulmonary artery banding (constriction) (N = 9; 4.3%). Such models were based on the single pathological insult (one-hit model) (total number of interventions: N = 185; 89.4%). Multi-hit models (10.6%) were presented in single studies; these included combinations of monocrotaline with exposure to a high-fat diet (MCT + HFD), use of the VEGF receptor blocker SU5416 followed by chronic hypoxia (SU5416+CH) or in ZSF1 Obese rats (ZSF1 Obese + SU5416). Most cases used rat models (75.5%): Wistar (47.9%) and Sprague-Dawley (SD) (38.5%). Others used mice, with most performed on C57BL (31.1%). Most protocols (91.3%) concerned male subjects, only. The median age of the animals at the beginning of study was 8 weeks (IQR, 6–10). More detailed information about the studies included in the analysis is provided in Supplementary Table S3.

### 3.3 LVH models and their potency to develop PH

Table 1 summarizes the most common animal models evaluated in the reviewed study protocols, divided into one- and multi-hit approaches. It presents the effects of these approaches on the development of pulmonary hypertension with special attention to hemodynamic parameters (RVSP, mPAP, CO) as well as PVR, RV remodeling, hypertrophy and fibrosis, and PA muscularization.

**TABLE 1:** Animal models of pulmonary hypertension accompanied with LV failure.

Backgro-und	Model	Species	Median time (wks)	Pulmonary hypertension	Left heart failure						
				RV hemodynamics	RV and (or) PA remodeling	Systolic function	Diastolic function	Lung mass	LVH	LV fibrosis	BW*
Single-hit											
Common PH	Monocrotaline inj. (MCT)	rat	4	↑↑RVSP; ↑↑mPAP; ↓CO; ↑PVR	↑↑RVH; ↑↑RV wall thickness; ↑↑PA remodeling	↓EF% (*p* = 0.097); ↔d*P*/d*t*_max_; ↔systolic (overall); ↓BP	↓d*P*/d*t*_min_; ↔ LVEDP; ↔diastolic (overall)	↑	↑	↑	↓
	Chronic hypoxia (CH)	rat/mouse	4	↑RVSP; ↑mPAP; ↓CO; ↑PVR	↑↑RVH; ↑↑RV wall thickness; ↑PA remodeling	↔EF%	↑LVEDP; ↔ diastolic (overall)	↑	↔		↓
	PA banding (PAB)/PA constriction (PAC)/pulmonary trunk banding (PTB)	rat/mouse	4	↑↑RVSP; ↓CO	↑↑RVH; ↑RV wall thickness	↔EF%; ↔systolic (overall)	↔LVEDd; ↔diastolic (overall)		↔	↑	↔
Pressure-overload HF	Aortic banding (AB)	rat/guinea pig	6	↑RVSP; ↑mPAP; ↑PVR	↑RVH; ↑RV wall thickness; ↑↑PA remodeling	↔EF%; ↓d*P*/d*t*_max_; ↑LVESd	↓d*P*/d*t*_min_; ↑LVEDP; ↔LVEDd	↑	↑	↑	↓
	Supracoronary aortic banding (SAB)	rat	9	↑mPAP; ↑PVR	↑↑RVH; ↑↑PA remodeling	↑LVSP; ↓systolic (overall)	↑LVEDP; ↓diastolic (overall)		↑	↑	↔
	Transverse aortic constriction (TAC)	rat/mouse	4.5	↑RVSP; ↑PVR; ↓CO	↑RVH; ↑RV wall thickness; ↑PA remodeling	↓d*P*/d*t*_max_; ↓EF%; ↑LVSP; ↓systolic (overall)	↓d*P*/d*t*_min_; ↑LVEDP; ↔LVEDd; ↓diastolic (overall)	↑	↑↑	↑	↔
	Left atrial stenosis (LAS)	rat	9	↓CO	↑RVH	↔EF%	↑LVEDP	↑			
Ischemic HF	Ligation of left coronary artery (myocardial infarction − MI)	rat/mouse	4	↑RVSP; ↑PVR	↑↑RVH; ↑PA remodeling	↓d*P*/d*t*_max_; ↓EF%; ↓LVSP; ↑LVESd; ↓systolic (overall)	↓d*P*/d*t*_min_; ↑LVEDP; ↑LVEDd; ↓diastolic (overall)	↑	↑	↑	↓
Metabolic (obesity)-HF	High-fat diets (HFD)	rat/mouse	20	↑RVSP; mPAP; ↔CO; ↔PVR	↑RVH; ↑RV wall thickness; ↑PA remodeling	↔EF%; ↔systolic (overall)	↑LVEDP (*p* = 0.084)	↑	↑		↑
	ZSF1 Obese	rat	8	↔RVSP	↔RVH	↔EF%; ↔LVSP; ↔systolic (overall)	↑LVEDP				
Systolic-HF	Dahl salt-sensitive	rat	8		↔RVH; ↑PA remodeling	↑BP			↑↑		
	SHRSP	rat	7	↑mPAP	↑PA remodeling; ↔RVH			↑	↑		↓
Multi-hit											
	ApoE^−/−^ + HFD	mouse	10	↔CO; ↑RVSP	↑RVH; ↑PA remodeling	↔EF%					
	Monocrotaline inj. (MCT) + HFD	rat	7	↑RVSP; ↓CO		↔EF%					↓
	SU5416+chronic hypoxia (+normoxia)	rat/mouse	3.2	↑↑RVSP; ↑mPAP; ↓CO	↑↑RVH; ↑↑RV wall thickness; ↑↑ PA remodeling	↔EF%; ↔ LVSP; ↔d*P*/d*t*_max_; ↔systolic (overall)	↔d*P*/d*t*_min_; ↔diastolic (overall)	↑	↔		↔
	Supracoronary aortic banding (SAB)+HFD	rat	9	↑mPAP; ↑PVR	↑PA remodeling	↔EF%; ↑LVSP	↑LVEDP		↑		
	ZSF1 Obese + SU5416	rat	11	↑RVSP; ↑PVR	↑RVH	↔EF%; ↔LVSP; ↔systolic (overall)	↑LVEDP				↑

**FIGURE 4:**
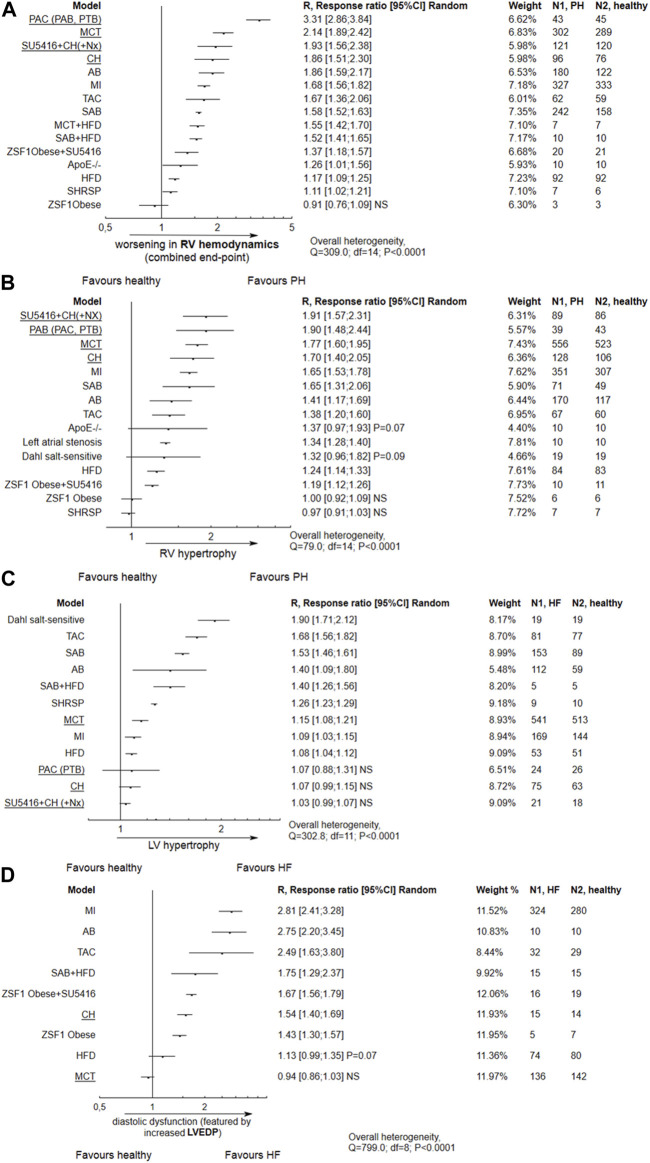
Alterations in right ventricular (RV) and left ventricular (LV) performance according to different animal models. In most tested models, apart from ZSF1 Obese rats, tree plots for the effect size expressed as R (response ratio) indicate the following significant trends: **(A)** Impaired RV hemodynamics with a composite end-point including an increase in right ventricular systolic pressure (RVSP), mean pulmonary arterial pressure (mPAP) and pulmonary vascular resistance (PVR); **(B)** RV hypertrophy, observed in most models except ZSF1 Obese and stroke-prone spontaneously hypertensive rats; **(C)** LV hypertrophy observed particularly in Dahl salt-sensitive rats, animals with (supracoronary) aortic banding (AB, SAB), transverse aortic constriction (TAC) or stroke-prone spontaneously hypertensive rats (SHRSP); **(D)** Diastolic dysfunction characterized by increased left ventricular end-diastolic pressure (LVEDP), observed in most tested models apart from MCT-treated subjects. NS,non significant. An effect size (R) > 1 indicates an increase in the mean value of a parameter in particular animal model as compared to healthy subjects, and worsening in PH/LVF, e.g., R = 1.90 would indicate an approximately two-fold increase. Common PH models are underlined. The effect size was calculated according to Eqs 2, 4.

Alterations in right ventricle (RV) hemodynamic parameters and RV hypertrophy are given in Figures 4A, B. The majority of models, except from the one-hit ZSF1 Obese rats, demonstrated worsening in RVSP, mPAP or PVR parameters (composite end-point). The most pronounced alterations were demonstrated for the common PH models, e.g., monocrotaline, pulmonary artery banding or combination of SU5416 and chronic hypoxia, but were also seen in animals with LV failure induced by ischemic conditions (myocardial infarction, MI) or pressure overload (aortic banding). A similar tendency was observed for RV hypertrophy.

Worsened RV hemodynamics and hypertrophy, as well as PA remodeling, were observed in relation to the body weight gain in metabolic models: HFD or ZSF1 Obese + SU5416 (Supplementary Figure S1). The changes in final BW according for each model are presented in Supplementary Table S4.

**FIGURE 5:**
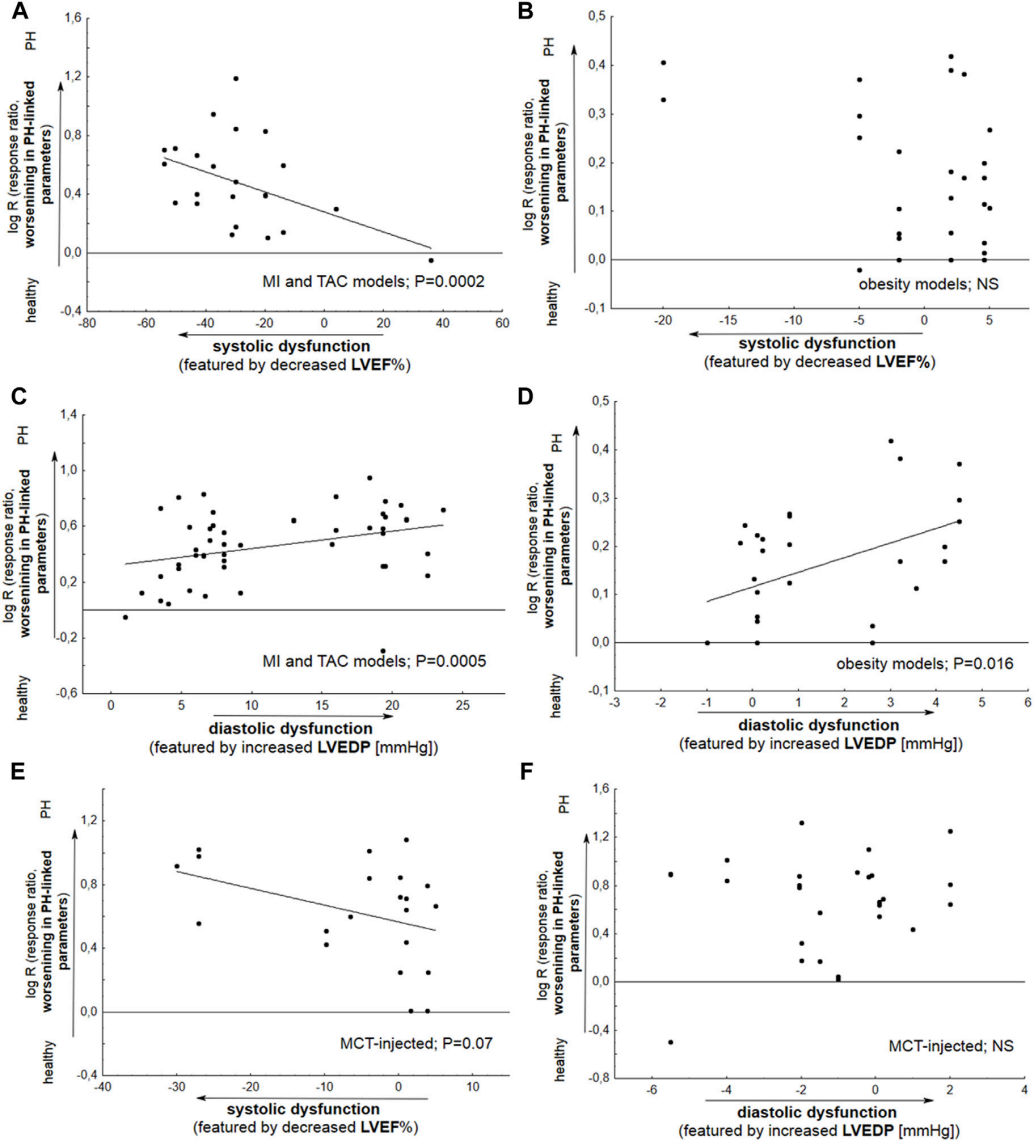
Meta-regression plots demonstrate the changes in particular parameters associated with pulmonary hypertension (RVSP, mPAP, PVR, RVH, and PA remodeling) as a function of LVEF% **(A,B,E)** or LVEDP **(C,D,F)**. Alterations in LVEF% (LVEDP) were expressed as Δ LVEF% (LVEDP), reflecting differences in the mean values between Vehicle and healthy subjects. **(A)** A significant deterioration (*p* = 0.0002) in PH-linked parameters was found to be accompanied by decreased LV ejection fraction in animals with coronary artery ligation (myocardial infarction, MI) or transverse aortic constriction (TAC); **(B)** Metabolic (obesity) models do not demonstrate any relationship between systolic dysfunction and PH worsening (*p* > 0.05); **(C,D)** Significant relationships were noted between deterioration in PH-linked parameters and increased LVEDP based on the MI and TAC models (*p* = 0.0005) and by metabolic models (*p* = 0.016); **(E)** Monocrotaline (MCT) injected animals reveal slight deterioration (*p* = 0.07) of PH due to decrease in LVEF%; **(F)** No such relationship was noted for MCT (*p* > 0.05) (N = 105 interventions). The effect size was calculated according to Eqs 2, 4.

The impairment in hemodynamic and hypertrophic PH-linked parameters was significantly correlated with the degree of reduction of LV ejection fraction, indicating systolic dysfunction. This was observed for HF models based on the coronary artery ligation and transverse aortic constriction (*p* = 0.0002). In addition, TAC and ischemic models demonstrated more pronounced diastolic dysfunction, as featured by increased LVEDP (*p* = 0.0005). Metabolic (obesity) approaches, *viz.* HFD and ZSF1 Obese + SU5416, do not reveal any relationship between systolic dysfunction and worse pulmonary hypertension (*p* > 0.05). However, PH was developed with more pronounced diastolic dysfunction in the latter models (Figures 5A–D).

### 3.4 The potential of common PH models to induce LV failure

A series of features of LV failure, i.e., systolic function (systolic blood pressure, LVEF%, d*P*/d*t*_max_, LVSP), active diastolic relaxation (d*P*/d*t*_min_), passive stiffness (LVEDP), LV structure (LVH), LV morphology (LVESd, LVEDd) and LV fibrosis, are presented in relation to the most commonly-used evaluated animal models in Table 1. The mean left ventricular ejection fraction (LVEF%) values are presented according to particular animal models of PH in Supplementary Table S5. In animals exposed to coronary artery ligation or transverse aortic constriction, the mean LV ejection fraction was significantly reduced and below the 50% threshold, i.e. 42.52% (95% CI 32.79–52.25), and 40.83% (95% CI 26.57–55.09).

As demonstrated in Figure 4C, none of the common models of PH except for monocrotaline injection demonstrated significant left ventricular hypertrophy. In contrast, LV hypertrophy was promoted by models of LV heart failure, such as high salt diet given to Dahl salt-sensitive subjects, and by transverse aortic constriction, or aortic (supracoronary) aortic banding. Diastolic dysfuntion, featured by increased LVEDP, was observed in most models except MCT-treated animals (Figure 4D).

In subjects injected with monocrotaline, worse PH was slightly correlated with systolic dysfunction featured by ↓LVEF% (*p* = 0.07). No correlation was demonstrated between increase in a parameter of diastolic dysfunction—LVEDP, and progression of pulmonary hypertension (Figures 5E–F).

**FIGURE 6:**
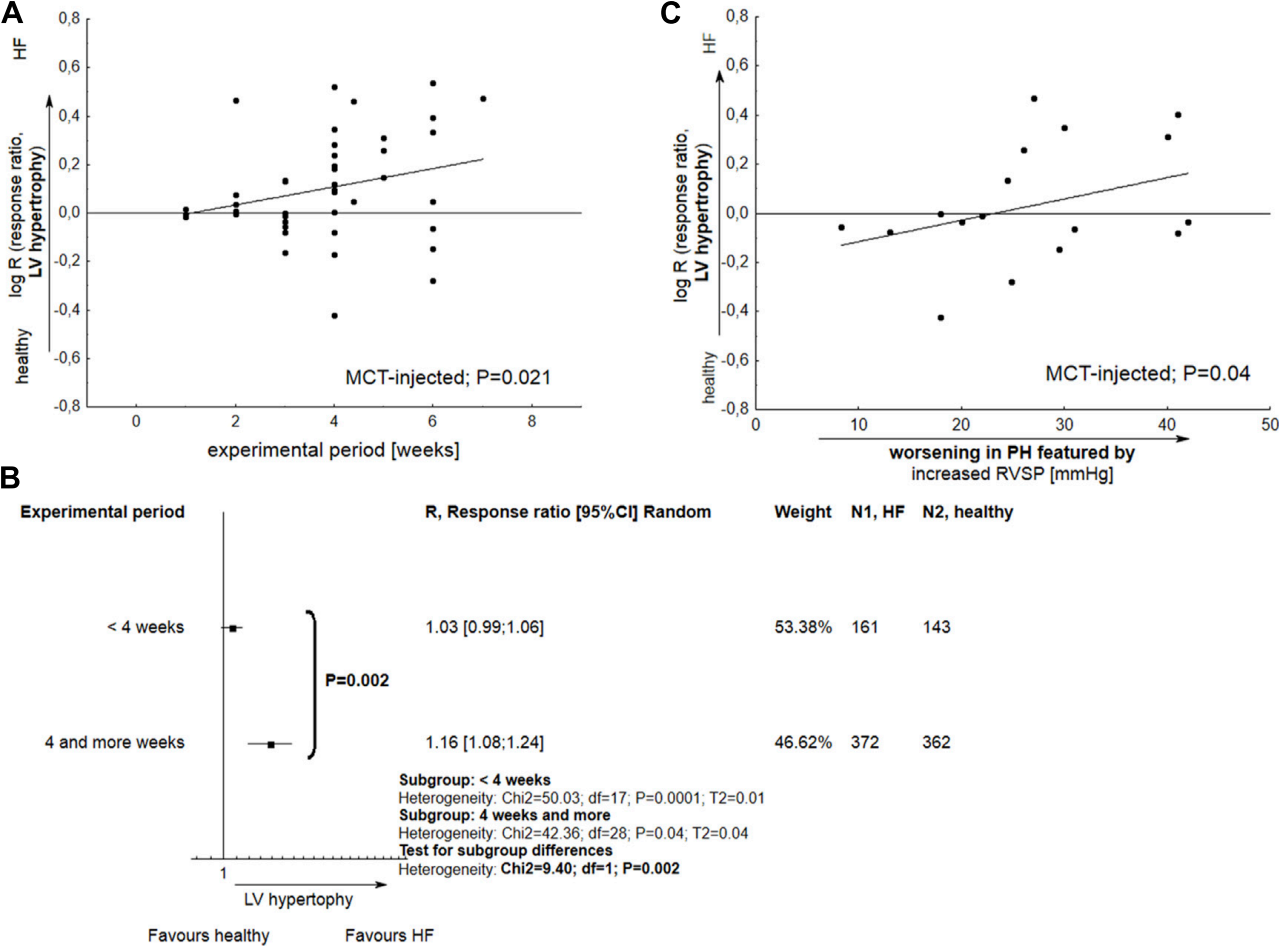
The performance of left ventricular hypertrophy (LVH) in monocrotaline injected rats. **(A)** Meta-regression plot demonstrates the alterations in LVH as a function of experimental period; **(B)** Tree-plot demonstrates the comparison of LV hypertrophy in experiments lasting <4 or >=4 weeks; **(C)** The significant development of LVH is accompanied with increased right ventricular systolic pressure. Increase in RVSP was expressed as Δ RVSP, indicating difference in mean values between Vehicle and healthy subjects (N = 47 interventions). The effect size was calculated according to Eqs 2, 4.

The development of LV hypertrophy in MCT-treated subjects was more pronounced for longer experiments, i.e., those lasting 4 weeks or more (*p* < 0.05). A slight relationship was also observed between the deterioration of LV hypertrophy and a worsening in RVSP—considered a marker for poor clinical outcomes in PH (*p* = 0.04) (Figure 6).

### 3.5 Animal survival

**FIGURE 7:**
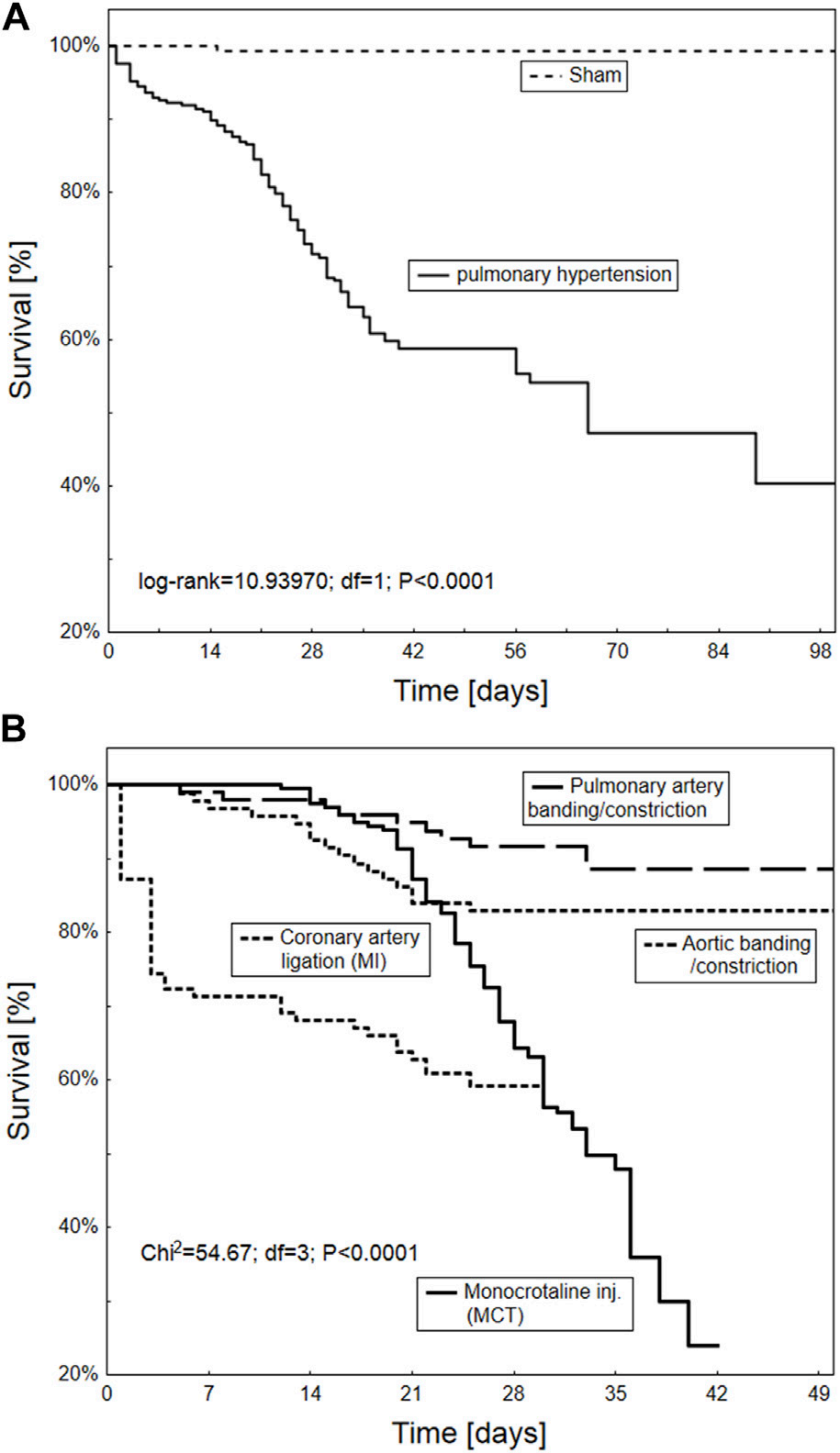
Kaplan-Meier survival curve of the overall survival according to different animal models of pulmonary hypertension overall **(A)** and in particular procedures including PA banding/constriction, MCT injection, aortic banding/constriction and coronary artery ligation **(B)** (*n* = 665 animals).

The Kaplan-Meier survival curve indicates significantly worse overall survival in animals with common PH or due to LVH compared to healthy subjects (*p* < 0.0001). In particular, animal models based on MCT injection or coronary artery ligation demonstrated six to 7-week poorer survival (Figure 7).

## 4 Discussion

The first finding of current systematic review is that the approaches intended to promote LV failure may also have the potential to develop pulmonary hypertension, with impaired RV hemodynamics as well as RV hypertrophy. This was true especially for aortic banded subjects, these with transverse aortic constriction (TAC), or myocardial infarction (MI) due to coronary artery ligation. For example, the mean increase in RVSP was 11.90 mmHg (95% CI 5.28–18.52) in animals undergoing aortic banding (ascending, abdominal), 15.12 mmHg (95% CI 8.57–21.66) for ligation of transverse aorta, and 17.69 mmHg (95% CI 14.40–20.98) for ligation of coronary artery. The following sections will describe and compare the efficacy of the most popular approaches addressing PH-LHD.

### 4.1 Pulmonary hypertension and ischemic heart failure or due to pressure overload

Methods that attempt to provoke PH by inducing ischemic heart failure have the advantage of replicating human PH due to myocardial cell necrosis and HF. Although the current study did not focus directly on the assessment of LV-related parameters in common LVH models, current findings confirm that coronary artery ligation can induce heart failure with reduced ejection fraction (HFrEF). This procedure resulted in significant diastolic dysfunction (↑LVEDP) and decreased LVEF% (below 50%) accompanied by significant impairments in systolic function, indicated by d*P*/d*t*_max_, LVSP, and LV morphometric changes (LVESd). Similar observations were made for transverse aortic constriction (TAC), another common model for pressure-load-induced heart failure, confirming that both models could increase the risk of left heart failure and death. The promotion of PH was revealed to be significantly correlated with the degree to which ischemic-HF and TAC models provoke such LV failure-related features. As demonstrated in the present paper, a worsening in RV hemodynamic, RV hypertrophy and PA remodeling was accompanied by pronounced left ventricular diastolic and systolic dysfunction, featured by augmented LVEDP and decreased LVEF%. Subjects undergoing transverse aortic constriction can experience sudden elevations in LV pressure and acute left heart failure; this phenomenon has been observed mainly in older mice. The disadvantages associated with ischemic-HF models are difficulties in controlling the area of infarct size, and poor animal survival due to cardiac arrhythmias, bleeding or pneumothorax (Breitling et al., 2015; Liu and Yan, 2022). Such increased animal mortality is also confirmed by the current analysis and might be recognized as an obstacle for research on PH.

Steady progression of cardiac dysfunction with PH under chronic pressure overload can be also provided by the aortic banding (AB) model. Aortic banding is a simple and reproducible procedure, and stimulates heart failure with preserved ejection fraction. This phenomenon was confirmed in the present study by “preserved” LVEF% (74.59%, 95% CI 72.86–76.33), and raised LVEDP, a parameter that features diastolic dysfunction. The development of pulmonary hypertension by aortic banding, like transverse aortic constriction, is uncommon in humans, and this could represent a limitation of the model for replicating human PH-LHD (Breitling et al., 2015; Liu and Yan, 2022).

In recent years, another model of PH from pressure overload-induced LV failure was proposed by Fujimoto et al. (2017). Left atrial stenosis (LAS) surgery could stimulate pulmonary venous arterialization and PH development due to mitral stenosis. Some worsening in RV hypertrophy accompanied by decrease in cardiac output were observed. However, due to limited number of studies included into current analysis, the results regarding the worsening of PH cannot be conclusive.

### 4.2 Pulmonary hypertension and metabolic models of heart failure

As introduced above, more than 50% of all patients with heart failure exhibit a large number of comorbidities, including hypertension, diabetes mellitus (DM) and/or obesity affecting the entire cardiovascular system (Gevaert et al., 2022). These patients are commonly classified to HF with preserved ejection fraction (HFpEF), where diastolic dysfunction exists in the presence of normal (near-normal) systolic LV performance. The development of PH in HFpEF has been increasingly recognized as clinical complication of metabolic syndrome and hence, most patients with PH-LHD also present with metabolic syndrome (Ussavarungsi et al., 2017). A very recent paper evaluates more than 500 protocols covering a wide range of systemic hypertension and diabetes (obesity)-related animal models proposed for preclinical studies on heart failure, especially HFpEF (Jasińska-Stroschein, 2022). In contrast, in the current paper, only one of ten protocols was performed on rodents with HFpEF and PH (10.1%). This was due to the fact that animal models in which PH develops due to LV failure, related with metabolic disease, have been developed in the last few years. Even fewer studies evaluate exercise intolerance as an important phenotypical characteristics of HFpEF, in addition to other features such as diastolic dysfunction, pulmonary congestion and concentric cardiac hypertrophy (Valero-Muñoz and Sam, 2017).

The reviewed protocols related to metabolic models of PH-LHD mainly concern the induction of heart failure by a high-fat diet, commonly due to chronic exposure to a 60% kcal fat diet, resulting in significant increase in body weight: mean increase 11.26 g (95% CI 8.83–13.66). The approach offers simplicity, convenience and noninvasiveness, but it requires prolongation of the experimental period. For example, the median length of the protocols included in the current analysis was 20 weeks. Another determinant of the HFD model could be animal race and strain. Meng et al. (2017) recently demonstrated that the AKR/J mouse more effectively developed RV systolic pressure, LVEDP, RV and LV hypertrophy, as well as glucose intolerance and elevated HbA1c levels, compared to C57BL/6J mice exposed to a high-fat diet. It has been proposed that the latter mouse model features metabolic syndrome, pulmonary hypertension and HFpEF in humans (Meng et al., 2017).

A very recent two-hit approach combined metabolic syndrome, as a consequence of obese ZSF1 with double–leptin receptor defect, and pulmonary endothelial impairment by the VEGF receptor antagonist, SU5416. A high-fat diet or the combination of ZSF1Obese + SU5416 provided a mild increase in rodent RVSP parameter (6.78 mmHg; 95% CI 3.69–9.87) and (9.25 mmHg; 95% CI 6.01–12.48), as well as mild RV hypertrophy. The models did not display alterations in LVEF% (systolic function), while diastolic function could be impaired, as indicated by increased LVEDP values. Note that exposure to SU5416 alone had no effect on the echocardiographic (hemodynamic) features of LVF, and obese ZSF1 rats did not develop PH. Diastolic dysfuntion was significantly correlated with a worsening in RV hemodynamics, RV hypertrophy and PA remodeling in this metabolic model of PH-LHD (PH-HFpEF).

As described above, dyslipidemia, glucose intolerance or diabetes can increase the likelihood of left heart disease as a cause of pulmonary hypertension (Humbert et al., 2023). There is also a high prevalence of obesity among patients with PH-LHD. Therefore, the preclinical studies included in the current analysis were tested as to whether increasing mass might determine PH-related lesions. This was true for HFD-treated subjects or ZSF1 animals injected with SU5416, where more pronounced PH was accompanied by body weight gain. A single study reported a correlation between hemodynamic features of PH-LHD and HbA1c levels in HFD-AKR/J mice. These results were consistent with previous clinical findings linking hyperglycemia and diabetes to increased LVEDP and elevated pulmonary pressures in patients with PH-HFpEF (Meng et al., 2017). All these phenomena suggest that the proposed metabolic approaches may be suitable for modeling pulmonary hypertension due to left heart disease, e.g., HFpEF. However, due to the limited number of protocols, further trials are needed.

Other two-hit approaches based on the combination of a high-fat diet with supracoronary aortic banding (SAB + HFD) or monocrotaline (MCT + HFD) have been studied. Both models have only been evaluated in single protocols; their findings indicate a significant worsening in PH-related parameters, with various impacts on systolic and diastolic LV function, making the obtained results inconclusive.

### 4.3 Pulmonary hypertension and systemic hypertensive models of heart failure

A history of hypertension is another risk factor for HFpEF that increases the likelihood of PH-LHD in elderly patients. Systemic hypertensive models of HF have been widely used for the purposes of LV failure-related preclinical studies. Dahl-salt sensitive, SHHF, or SHR, provide significant systolic function deterioration as evidenced by a decrease in LV d*P*/d*t*_max_, fractional shortening, and ejection fraction (Jasińska-Stroschein, 2022). An excessive rise in blood pressure has been found to significantly determine the severity of model. Systemic hypertensive models could demonstrate impaired diastolic performance, progression to systolic dysfunction, and transition from compensated hypertrophy to congestive heart failure influenced by *inter alia* experiment duration. However, evidence of the utility of systemic hypertensive models for studies on pulmonary hypertension remains poor. In the present analysis, very few PH-related experimental protocols concerned Dahl-salt sensitive rats exposed to a high-salt (8%) diet, or stroke-prone spontaneously hypertensive rats (SHRSP). The latter is a unique genetic model of severe hypertension and cerebral stroke (Nabika et al., 2012). Animals displayed mild worsening in RV hemodynamics, without RV hypertrophic lesions; however, due to limited data, the present characteristics of both models awaits further confirmation.

### 4.4 Common models of pulmonary hypertension and risk of left heart failure

As mentioned above, apart from evaluating the potency of common LVH models to promote PH-related lesions (*primary end-point*), an additional issue was to determine whether, and to what extent, common PH models could develop LV failure (*secondary end-point*). As expected, the majority of common PH rodent approaches developed pulmonary hypertension, as evidenced by alterations in RV hemodynamic parameters (↑ RVSP, mPAP and PVR; ↓ CO) as well as worsening in RV hypertrophy. The most pronounced changes were seen for pulmonary artery banding (constriction), SU5416 plus chronic hypoxia, and monocrotaline.

Among all common PH models, only monocrotaline-injected subjects revealed increase in LV hypertrophy (*p* < 0.05) and a mild decrease in LVEF%–a marker of systolic dysfunction (*p* = 0.097). In MCT-injected animals, LV hypertrophy was observed for longer experimental periods (*p* = 0.021), especially more than 4 weeks. LV hypertrophy was also more pronounced in subjects with elevated RVSP, a marker for poor clinical outcomes in PH (*p* = 0.04). Next, for the MCT approach, a slight (*p* = 0.07) correlation was observed between decrease in LVEF% and worsening in PH-related lesions. In almost all the studied papers, the mean LVEF% in MCT-treated rats was above 50% (63.82; 95% CI 55.78–71.86), with a single injection of MCT causing a pronounced decrease, i.e., below 40%, in only two studies (Piao et al., 2012; Alencar et al., 2017). Even so, the possible reason for this phenomenon remains unclear. Few papers have examined the potential detrimental impact of MCT on LV dysfunction. For example, Hołda et al. (2020) assessed changes in cardiac parameters at both the early and end-stage of pulmonary arterial hypertension (PAH). The authors conclude that myocarditis, with significant inflammatory infiltration, results in the increase in the size and weight of the left ventricle observed at the end stages of the disease. This phenomenon might concern approximately 15% of all monocrotaline injected rats, where left ventricular myocarditis developed at end-stage PAH but not in early PAH (Nogueira-Ferreira et al., 2015).

As expected, the monocrotaline model was characterized by a pronounced increase in animal mortality within a six-to 7-week period in current study. Poorer results were only observed for ischemic heart-failure induced PH. Monocrotaline (MCT) treatment is well known to be associated with poor animal survival. Its active toxic derivative, dehydromonocrotaline, forms in hepatic P450 3A cytochrome, and damages the endothelium of PA vessels; this causes pathological remodeling, with the subsequent development of pulmonary hypertension, hypertrophy of the right ventricle myocardium, and RV heart failure (Hołda et al., 2020). Even so, the MCT model seems to remain the most frequently chosen for the purposes of PH experiments.

Chronic hypoxia is a common rodent model featuring group 3 of human PH, i.e., associated with lung diseases and/or hypoxia. Compared to monocrotaline (group 1, pulmonary arterial hypertension), prolonged exposure to hypoxic conditions (10% oxygen) has been demonstrated to provoke “milder” hypertension in rodent pulmonary circulation, with insignificant impact on the LV hypertrophy and systolic function featured by LV ejection fraction. Interestingly, hypoxic animals displayed increased LVEDP values; this finding resembles clinical observations, where patients suffering from chronic obstructive pulmonary disease (COPD) with hypoxemia may develop LV diastolic dysfunction. The proposed mechanism for the impaired relaxation and diastolic dysfunction in both RV and LV might include changes in proteins governing removal of cytosolic Ca2+ in diastole, due to inhibition of SERCA and Ca2+ pump activity (Larsen et al., 2006). As mentioned above, hypoxic conditions did not affect other parameters related to left ventricular function and morphology.

### 4.5 Limitations

The study has several limitations. First the search criteria were limited to rodents, while some models, e.g., these based on the ligation of left coronary artery or pulmonary vein banding, could also address large animals. The effect size, expressed as risk ratio, allowed reducing inter-species differences (e.g., mice and rats). Another point was that only a limited number of experimental protocols included data about pulmonary congestion, and even fewer examined exercise capacity; both are important features for identification of HFpEF. For this reason, a full quantitative analysis of all factors that constitute PH-LHD in relation to HFpEF was difficult. Similarly, due to the limited number of experiments, the potential advantages of two-hit approaches, including, e.g., monocrotaline, high-fat diet, and supracoronary artery banding, require further estimation. Next, the heterogeneity between studies was generally high. Furthermore, the majority of experiments were performed with males. Therefore further studies are needed to evaluate whether a sex bias exists, with sex hormones determining the development of PH-LHD. Similar studies could have been focused on inter-strain differences in potential susceptibility to PH development, as indicated for the AKR/J and HFD model. Finally, due to the limited amount of data, it was not possible to test for correlations between changes in particular features of LV or RV dysfunction and glycemia, plasma insulin or triglycerides.

A key strength of the paper is that it was based on the results of a relatively large number of experimental protocols (*n* = 135 papers). Subgroup analyses were performed to reduce inter-study heterogeneity: to account for different experimental conditions, with special attention to animal model. The analyses addressed separate changes in RV and LV performance, featured by a variety of hemodynamic, echocardiographic, and histopathologic parameters. Meta-regression analyses were performed to evaluate the hypothesis about the potential correlation between the worsening in LV performance and PH development. Little publication bias was observed across the studies, and sensitivity analyses indicated, in most cases, no influence of the individual study on the final result, according to the leave-one-out method.

### 4.6 Further implications

Growing understanding of pulmonary hypertension caused by left heart disease (PH-LHD) has brought with it a need for further development of animal models that can replicate human disease. In the past, most preclinical studies on PH evaluated the potential efficacy of pharmacological agents used in humans for treatment of LV failure, e.g., beta-blockers (BBs) or renin-angiotensin-aldosterone system blockers (angiotensin converting enzyme inhibitors-ACEIs, mineralocorticoid receptor antagonists-MRAs). They were performed on common PH models based on monocrotaline (group 1, pulmonary arterial hypertension) or chronic hypoxia (group 3, PH associated with lung diseases and/or hypoxia) (van Suylen et al., 1998; de Man et al., 2012; Liu et al., 2021). Considering the complexity of the pathophysiological background of the disease, recent attempts have investigated multi-hit approaches, in which PH is induced by metabolic dysfunction due to high-fat diets or genetic modifications in combination with exposure to a VEGF blocker. Other studies have focused on animal models of pressure overload-induced HF and PH. Both models, i.e., these related with metabolic dysfunction, or with pressure overload, were used to explore the efficacy of sodium-glucose cotransporter 2 (SGLT-2) inhibitors in PH due to LV failure (Satoh et al., 2017; Joki et al., 2022). However, these findings have not been translated into clinical trials. This could be explained by the fact that, in addition to the differences between animal and human disease, the treatment of HFpEF itself remains challenging. Many recently-tested medical interventions, e.g., beta-blockers, ACEIs, MRAs, and angiotensin receptor/neprilysin inhibitors have failed to reduce primary endpoints in HFpEF patients in respective cardiovascular outcomes trials (Riccardi et al., 2022). Very recent observations from clinical trials have identified SGLT-2 inhibitors as the first class of drugs to improve prognosis in patients with HF and mildly reduced or preserved LVEF% (Vaduganathan et al., 2022), but this must be confirmed by further clinical evaluations. Interestingly, studies have been planned to investigate the effects of SGLT-2 inhibitors: dapagliflozin or empagliflozin on exercise capacity and hemodynamics in patients with pulmonary arterial hypertension (NCT05179356; NCT05493371).

The need for effective treatments has resulted in the development of a wide range of preclinical models of PH-LHD. Although their use has advanced understanding of the disorder, they require greater reproducibility and repeatability, and ensure good animal survival thorough the experiment. In detail, current findings confirm that:

- Aortic banded subjects, these with transverse aortic constriction (TAC), or myocardial infarction (MI) due to coronary artery ligation demonstrated more pronounced worsening in PH due to LV failure.
- The degree in which TAC and ischemic-HF models (MI) provoke PH-related features can be significantly correlated with promotion of systolic (decrease in LVEF%) and diastolic (increase in LVEDP) dysfunction. The latter approach (MI) might result in poor animal survival, however.
- Prolonged exposure to a high-fat diet or two-hit model of obese ZSF1 rat with pulmonary endothelial impairment by the VEGF receptor antagonist SU5416 might impair diastolic function and worsen pulmonary hypertension. These models could be promising options for studies on PH-LHD; however, due to the limited number of existing protocols, further investigations are needed.
- Among all common PH models, only monocrotaline-injected subjects revealed some increase in LV hypertrophy. Such changes were associated with longer study periods, especially when experiments lasted more than 4 weeks, as well as with worsened RV hemodynamics. In this model, the potential mechanism might include myocarditis at the end-stage of PH.
- The benefits of other two-hit approaches that combine common PH model and LV failure (e.g., MCT + HFD) should be evaluated in further preclinical trials.
