# Supplementary material for: An updated review of experimental rodent models of pulmonary hypertension and left heart disease
Source: Front Pharmacol. 2024 Jan 8;14:1308095. doi: 10.3389/fphar.2023.1308095 (PMC10800974; doi:10.3389/fphar.2023.1308095)
Supplement: Supplementary file 2 [file DataSheet1.DOCX]

Supplementary Material

**Methods.**

**Search strategy**

The databases (MEDLINE and WEB OF SCIENCE; from 01-01-1992 to 31-12-2022) were searched with no language restrictions using the following search terms in titles and abstracts:

(PULMONARY HYPERTENSION AND HEART FAILURE OR SYSTOLIC DYSFUNCTION OR DIASTOLIC DYSFUNCTION OR HFEF OR HFRF)

AND (ANIMAL OR MOUSE OR MICE OR RAT)

OR

(AGNIOTENSIN II INFUSION OR ANG II INFUSION OR ANG II INFUSED OR ANGIOTENSIN II INFUSED)

OR

(ALDOSTERONE INFUSION OR ALD INFUSED OR ALDOSTERONE INFUSED OR ALD INFUSION)

OR

(APOE OR APOLIPOPROTEIN E)

OR

(DAHL-SALT SENSITIVE OR DAHL RAT OR DAHL SALT OR DAHL SALT SENSITIVE )

OR

(DB/DB OR DB DB OR DB MOUSE OR DB MICE OR DIABETIC CARDIOMYOPATHY)

OR

(DOCA SALT OR DEOXYCORTICOSTERONE OR DOCA-SALT OR DOCA-SALT HYPERTENSION OR DOCA HYPERTENSION OR DOCA SALT HYPERTENSION)

OR

(HIGH FAT DIET OR HFD OR WESTERN DIET OF MICE HIGH FAT)

OR

(OB/OB OR OB OB OR OB MOUSE OR OB MICE OR OB OBESITY)

OR

(SHHF OR SPONTANEOUS HYPERTENSION-HEART FAILURE)

OR

(SHR OR SPONTANEOUS HYPERTENSIVE RAT)

OR

(STZ OR STREPTOZOTOCIN OR STZ DIABETES)

OR

(ZUCKER RAT OR ZUCKER OBESE OR ZSF1 OR ZUCKER FATTY OR ZDF OR ZUCKER DIABETIC FATTY OR ZUCKER DIABETIC)

OR

(MONOCROTALINE OR MCT OR CROTALINE)

OR

(SUGEN OR SU5416 OR SU-5416)

OR

(CHRONIC HYPOXIA OR HYPOXIC OR 10%OXYGEN)

OR

(PULMONARY ARTERY BANDING OR PULMONARY ARTERIAL BANDING OR PULMONARY ARTERY CONSTRICTION OR PAB OR PAC OR PULMONARY ARTERIAL CONSTRICTION OR PULMONARY TRUNK BANDING)

OR

(LEFT PNEUMONECTOMY)

**Methods**

**Data extraction**

The following data were recorded: rodent strain and species, age, sex, mortality, and characteristics of the animal model of pulmonary hypertension (inductor, dose, surgical procedure, experimental period). The extracted PH-linked outcome data included hemodynamic parameters: right ventricle systolic pressure [RVSP], mean pulmonary arterial hypertension [mPAP], pulmonary vascular resistance [PVR], cardiac output [CO], right ventricle (RV) hypertrophy: RV mass, Fulton index [RV/LV+S], RV/BW, RVH%, RV/LV, RV mass/tibia length [RV/TL], RV thickness, pulmonary artery (PA) vascular remodelling. Outcomes related with left heart failure also included LV hypertrophy featured by: LV mass, LV/BW or LV/TL; lung mass, lung weight/tibia length (TL), or lung wet/dry ratio; hemodynamic data: systolic blood pressure, left ventricular systolic pressure [LVSP], left ventricular end-diastolic pressure [LVEDP] and time needed for relaxation of 50% maximal left ventricular pressure to baseline [tau]. The analysis also included echocardiographic and morphometric data: LV fractional shortening [FS], LV ejection fraction [EF], maximal rate of pressure rise [dP/dtmax] and pressure fall [dP/dtmin], left ventricular end-systolic diameter [LVESd], left ventricular end-diastolic diameter [LVEDd]. Histopathological data: percentage of LV fibrosis were also extracted.

The mean (+/‒ SD, or +/‒SEM), and number of animals per group (n) were recorded. If the study protocol provided the range of subjects (e.g., 5–12), the lowest number was used, in situation where the number of animals at the end of the study was lacked, the initial number of subjects (at randomization) was used. Two independent reviewers (MJ-S, AK) searched the literature, extracted the data and performed quality assessment. This was done independently; any differences were resolved until consensus was reached.

**Data analysis**

Alterations in BW, echocardiographic and hemodynamic parameters, were analysed based on the

## (Equation 1) $\boldsymbol{D}\left( \boldsymbol{diff in means} \right)\boldsymbol{=(}\boldsymbol{X}_{\boldsymbol{PH-LHD}}\boldsymbol{) - (}\boldsymbol{X}_{\boldsymbol{healthy}}\boldsymbol{)}$, or

(Equation 3) $\boldsymbol{D}\left( \boldsymbol{diff in means} \right)\boldsymbol{=(}\boldsymbol{X}_{\boldsymbol{LVF}}\boldsymbol{) - (}\boldsymbol{X}_{\boldsymbol{healthy}}\boldsymbol{)}$

Increased D (difference in means) values indicate a rise in the value of a particular parameter in the Vehicle group as compared to healthy subjects.

Due to the considerable heterogeneity of the parameters used to express inter alia left ventricle hypertrophy, right ventricle hypertrophy and fibrosis, any differences between healthy animals and subjects with PH-LHD were calculated using

(Equation 2) $\boldsymbol{R(risk ratio) = (}\boldsymbol{X}_{\boldsymbol{PH-LHD}}\boldsymbol{) / (}\boldsymbol{X}_{\boldsymbol{healthy}}\boldsymbol{)}$,

(Equation 4) $\boldsymbol{R(risk ratio) = (}\boldsymbol{X}_{\boldsymbol{LVF}}\boldsymbol{) / (}\boldsymbol{X}_{\boldsymbol{healthy}}\boldsymbol{)}$.

Such approach allowed including different parameters (with different units), and being denoted in different species (rat, mouse), into one analysis. For example, this applied to RV hypertrophy that was reported by authors as RV mass, Fulton index, RV/BW or RVH%. Response ratio was also used to express the discrepancies among groups of animals in relation to the composite end-points (e.g., the combination of RVSP, mPAP, and PVR as a feature of pulmonary hypertension). A response ratio (R) of 1 indicates no differences between subjects with PH-LHD (Vehicle) and healthy animals (Sham), while values R<1 (R>1) indicate a decrease (or an increase) in a particular parameter in a respective animal model of PH-LHD, as compared to healthy subjects.

**Results**

**Risk of bias**

## In total, 54.0 percent of the protocols (73/135) stated that the animals were randomly submitted to the Vehicle and placebo control group. Any details concerning the allocation sequence were not provided (unclear risk of bias). The remaining protocols did not provide any statement about the process of randomization (unclear risk of bias).

## Also 21.0 percent of the protocols (28/135) mentioned blinding at any level. However, although they reported that the assessment of outcome was blinded (mainly histomorphometric and statistical analyses), it was not stated how personnel and outcome assessors were blinded (unclear risk of bias). The remaining protocols did not give any statement about the blinding the animal caregivers and investigators (unclear risk of performance and detection bias).

The baseline characteristics of the animals was given in 18.0 percent of the protocols (low risk of bias). The assessments of completeness of outcome data, for each main outcome, should include the numbers of animals in each intervention and control group as compared with total randomized animals. Only in 45 out of 135 papers the number of animals per group at randomization and at the end of the study (low risk of bias) was given. In the remaining papers, data concerning the alterations of particular parameters were provided for the entire range of subjects (unclear risk of bias) and it was not possible to assess the final number of animals et the end of the experiment.


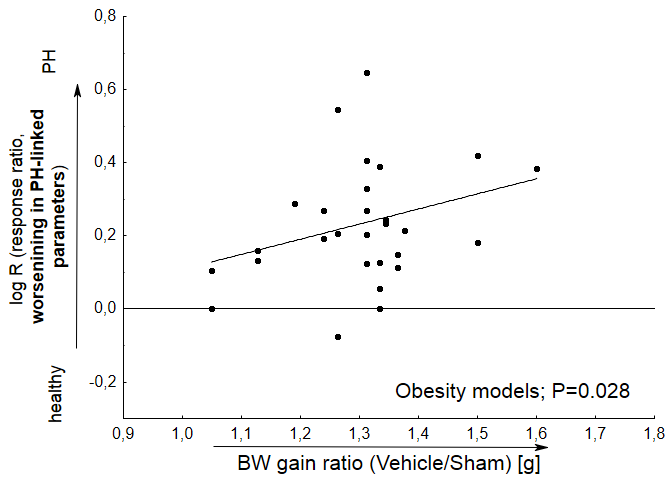


**Supplementary Figure 1.** The impact of BW gain on the RV hemodynamics, RVH and PA remodeling in metabolic models: HFD-treated subjects or ZSF1 Obese+SU5416. Bubble plot with fitted meta-regression line of effect size (log R) demonstrates the worsening in PH-related parameters – combined endpoint (RVSP, PVR, mPAP, RVH, PA muscularization) (P=0.028) (N=29 interventions).

**Supplementary Table 1.** Results from publication bias funnel plot and ‘trim and fill analysis’

| Animal model | Parameter (effect size) | Egger regression  P-value | Imputed (trim and fill) |
| --- | --- | --- | --- |
| All models | CO (D, difference in means) | P=0.0003 | 0 |
|  | RVSP (D, difference in means) | P=0.0005 | 0 |
|  | mPAP (D, difference in means) | P<0.0001 | 0 |
|  | PVR (D, difference in means) | P>0.05 | 11 |
|  | RV wall thickness (R, response ratio) | P>0.05 | 0 |
|  | RVH (R, response ratio) | P>0.05 | 0 |
|  | PA remodeling(R, response ratio) | P=0.001 | 0 |
|  | EF (D, difference in means) | P>0.05 | 0 |
|  | d*P*/d*t*_tmax_ (D, difference in means) | P>0.05 | 0 |
|  | LVSP (D, difference in means) | P>0.05 | 0 |
|  | LVESd (D, difference in means) | P>0.05 | 0 |
|  | LVEDd (D, difference in means) | P>0.05 | 0 |
|  | LVEDP (D, difference in means) | P>0.05 | 0 |
|  | d*P*/d*t*_tmin_ (D, difference in means) | P>0.05 | 0 |
|  | Lung mass (R, response ratio) | P=0.0023 | 0 |
|  | LVH (R, response ratio) | P>0.05 | 0 |
|  | Fibrosis (R, response ratio) | P=0.004 | 1 |
| Aortic banding (AB) | RVSP (D, difference in means) | P>0.05 | 0 |
| Chronic hypoxia |  | P>0.05 | 0 |
| High-fat diets (HFD) |  | P>0.05 | 0 |
| Ligation of left coronary artery (MI) |  | P>0.05 | 0 |
| Monocrotaline inj. (MCT) |  | P>0.05 | 1 |
| PA banding (PAB)/PA constriction (PAC)/pulmonary trunk banding (PTB) |  | P>0.05 | 0 |
| SU5416+chronic hypoxia (+normoxia) |  | P>0.05 | 0 |
| Transverse aortic constriction (TAC) |  | P>0.05 | 0 |
| ZSF1 Obese +SU5416 |  | P>0.05 | 0 |
| Aortic banding (AB) | mPAP (D, difference in means) | P>0.05 | 0 |
| Chronic hypoxia |  | P>0.05 | 0 |
| Monocrotaline inj. (MCT) |  | P>0.05 | 0 |
| Supracoronary aortic banding (SAB) |  | P>0.05 | 0 |
| SU5416+chronic hypoxia (+normoxia) |  | P>0.05 | 0 |
| Aortic banding (AB) | RVH (R, response ratio) | P=0.019 | 0 |
| Chronic hypoxia |  | P>0.05 | 0 |
| Dahl salt-sensitive |  | P>0.05 | 0 |
| High-fat diets (HFD) |  | P>0.05 | 0 |
| Ligation of left coronary artery (MI) |  | P>0.05 | 3 |
| Monocrotaline inj. (MCT) |  | P=0.04 | 0 |
| PA banding (PAB)/PA constriction (PAC)/pulmonary trunk banding (PTB) |  | P>0.05 | 0 |
| SU5416+chronic hypoxia (+normoxia) |  | P>0.05 | 0 |
| Supracoronary aortic banding (SAB) |  | P>0.05 | 0 |
| Transverse aortic constriction (TAC) |  | P=0.022 | 0 |
| ZSF1 Obese+SU5416 |  | P>0.05 | 0 |
| Aortic banding (AB) | PA remodeling(R, response ratio) | P>0.05 | 0 |
| High-fat diets (HFD) |  | P>0.05 | 0 |
| Ligation of left coronary artery (MI) |  | P>0.05 | 0 |
| Monocrotaline inj. (MCT) |  | P>0.05 | 0 |
| SU5416+chronic hypoxia (+normoxia) |  | P>0.05 | 0 |
| Supracoronary aortic banding (SAB) |  | P>0.05 | 0 |
| Transverse aortic constriction (TAC) |  | P>0.05 | 0 |
| Monocrotaline inj. (MCT) | RV wall thickness (R, response ratio) | P>0.05 | 0 |
| SU5416+chronic hypoxia (+normoxia) |  | P>0.05 | 0 |
| Aortic banding (AB) | EF (D, difference in means) | P>0.05 | 0 |
| High-fat diets (HFD) |  | P>0.05 | 0 |
| Ligation of left coronary artery (MI) |  | P>0.05 | 0 |
| Monocrotaline inj. (MCT) |  | P>0.05 | 1 |
| SU5416+chronic hypoxia (+normoxia) |  | P>0.05 | 0 |
| Transverse aortic constriction (TAC) |  | P>0.05 | 0 |
| High-fat diets (HFD) | LVEDP (D, difference in means) | P>0.05 | 0 |
| Ligation of left coronary artery (MI) |  | P=0.028 | 0 |
| Monocrotaline inj. (MCT) |  | P>0.05 | 1 |
| Transverse aortic constriction (TAC) |  | P>0.05 | 0 |
| ZSF1 Obese+SU5416 |  | P>0.05 | 0 |
| High-fat diets (HFD) | LVSP (D, difference in means) | P>0.05 | 0 |
| Ligation of left coronary artery (MI) |  | P>0.05 | 0 |
| Monocrotaline inj. (MCT) |  | P>0.05 | 0 |
| SU5416+chronic hypoxia (+normoxia) |  | P>0.05 | 0 |
| Transverse aortic constriction (TAC) |  | P>0.05 | 0 |
| Ligation of left coronary artery (MI) | LVESd (D, difference in means) | P>0.05 | 0 |
| Monocrotaline inj. (MCT) |  | P>0.05 | 0 |
| Transverse aortic constriction (TAC) |  | P>0.05 | 0 |
| Aortic banding (AB) | LVEDd (D, difference in means) | P>0.05 | 0 |
| Ligation of left coronary artery (MI) |  | P>0.05 | 0 |
| Monocrotaline inj. (MCT) |  | P>0.05 | 0 |
| Transverse aortic constriction (TAC) |  | P>0.05 | 0 |
| Aortic banding (AB) | LVH (R, response ratio) | P>0.05 | 0 |
| Chronic hypoxia |  | P>0.05 | 3 |
| Dahl-salt sensitive |  | P>0.05 | 3 |
| High-fat diets (HFD) |  | P=0.048 | 1 |
| Ligation of left coronary artery (MI) |  | P>0.05 | 0 |
| Monocrotaline inj. (MCT) |  | P>0.05 | 0 |
| PA banding (PAB)/PA constriction (PAC)/pulmonary trunk banding (PTB) |  | P>0.05 | 0 |
| SU5416+chronic hypoxia (+normoxia) |  | P>0.05 | 0 |
| Supracoronary aortic banding (SAB) |  | P>0.05 | 0 |
| Transverse aortic constriction (TAC) |  | P>0.05 | 0 |
| Ligation of left coronary artery (MI) | d*P*/d*t*_max_ (D, difference in means) | P>0.05 | 0 |
| Monocrotaline inj. (MCT) |  | P>0.05 | 0 |
| SU5416+chronic hypoxia (+normoxia) |  | P>0.05 | 0 |
| Transverse aortic constriction (TAC) |  | P>0.05 | 0 |
| Ligation of left coronary artery (MI) | d*P*/d*t*_min_ (D, difference in means) | P>0.05 | 0 |
| Monocrotaline inj. (MCT) |  | P>0.05 | 0 |
| SU5416+chronic hypoxia (+normoxia) |  | P>0.05 | 0 |
| Transverse aortic constriction (TAC) |  | P>0.05 | 0 |
| Aortic banding (AB) | Lung mass (R, response ratio) | P>0.05 | 0 |
| Chronic hypoxia |  | P>0.05 | 0 |
| Ligation of left coronary artery (MI) |  | P=0.03 | 0 |
| Monocrotaline inj. (MCT) |  | P>0.05 | 1 |
| Transverse aortic constriction (TAC) |  | P>0.05 | 0 |
| Aortic banding (AB) | BW (D, difference in means) | P>0.05 | 0 |
| Chronic hypoxia (CH) |  | P>0.05 | 0 |
| High-fat diets (HFD) |  | P>0.05 | 0 |
| Ligation of left coronary artery (MI) |  | P>0.05 | 2 |
| Monocrotaline inj. (MCT) |  | P>0.05 | 0 |
| Monocrotaline inj. (MCT) + HFD |  | P>0.05 |  |
| Pulmonary artery banding (PAB)/pulmonary artery constriction (PAC)/pulmonary trunk banding (PTB) |  | P>0.05 | 0 |
| Transverse aortic constriction (TAC) |  | P>0.05 | 1 |
| ZSF1 Obese+SU5416 |  | P>0.05 | 0 |

BW – body weight [g]; CO – cardiac output [ml/min]; d*P*/d*t*_max_ – maximal rate of pressure rise [mmHg/sec]; d*P*/d*t*_min_ –maximal rate of pressure fall [mmHg/sec]; EF – ejection fraction [%]; HFD – high fat diet; LVEDd – left ventricular end-diastolic dimension [mm]; LVEDP – left ventricular end-diastolic pressure [mmHg]; LVESd – left ventricular end-systolic dimension [mm]; LVH – left ventricular hypertrophy; LVSP – left ventricular systolic pressure [mmHg]; MI – myocardial infarction; mPAP – mean pulmonary arterial pressure [mmHg]; PA – pulmonary artery; PVR – pulmonary vascular resistance [mmHg/ml/min]; RV – right ventricle; RVSP – right ventricle systolic pressure [mmHg]; RVH – right ventricle hypertrophy; SHRSP – stroke-prone spontaneously hypertensive rat; TAC – transverse aortic constriction

**Supplementary Table 2.** Leave-one-out sensitivity analysis – the summary.

| Parameter  (effect size) | Item | Mean | 95% CI | P | Change in standard error* [Ref] | No of trials /interventions** |
| --- | --- | --- | --- | --- | --- | --- |
| RVSP  [mmHg] (D, difference in means) | Overall effect | 20.84 | 18.46−23.21 | <0.00001 | 0.00% | 98 |
|  | Statistics with study removed | 20.95 | 18.33−23.58 | <0.00001 | 10.55% [133] | – |
|  |  | 20.43 | 18.23–22.62 | <0.00001 | -7.42% [71] | – |
| mPAP [mmHg] (D, difference in means) | Overall effect | 12.28 | 11.12−13.44 | <0.00001 | 0.00% | 36 |
|  | Statistics with study removed | 12.96 | 11.04−14.87 | <0.00001 | 64.71% [51] | – |
|  |  | 11.70 | 10.68–12.72 | <0.00001 | -12.34% [19] | – |
| RVH  (R, response ratio) | Overall effect | 1.59 | 1.52–1.67 | <0.00001 | 0.00% | 158 |
|  | Statistics with study removed | 1.59 | 1.51–1.69 | <0.00001 | 19.54% [81] | – |
|  |  | 1.58 | 1.52–1.65 | <0.00001 | -16.26% [41] | – |
| EF [%] (D, difference in means) | Overall effect | -6.55 | -10.68−(-2.42) | =0.002 | 0.00% | 60 |
|  | Statistics with study removed | -6.64 | -11.54–(-1.72) | =0.008 | 18.88% [133] | – |
|  |  | -6.09 | -9.66–(-2.51) | =0.0008 | -13.45% [24] | – |
| LVEDd [mm]  (D, difference in means) | Overall effect | 0.10 | -0.46–0.66 | P>0.05 | 0.00% | 26 |
|  | Statistics with study removed | 0.22 | -0.31–0.75 | P>0.05 | -5.10% [68] | – |
|  |  | 0.06 | -0.56–0.68 | P>0.05 | 10.06% [51] | – |
| LVESd [mm]  (D, difference in means)  LVESd [mm] –  1 study was removed [11] | Overall effect  Statistics with study removed | 0.89 | 0.04–1.74 | P=0.04 | 0.00% | 19 |
|  |  | 0.91 | -0.078–1.89 | P>0.05 | 15.81% [133] | – |
|  |  | 0.64 | -0.13–1.42 | P>0.05 | -8.84% [57] | – |
|  | Overall effect | 0.64 | -0.17–1.45 | P>0.05 | 0.00% | 18 |
|  | Statistics with study removed | 0.65 | -0.30–1.60 | P>0.05 | 16.84% [133] | – |
|  |  | 0.37 | -0.35–1.08 | P>0.05 | -12.34% [57] | – |
| LVEDP [mmHg] (D, difference in means) | Overall effect | 5.61 | 4.40−6.83 | <0.00001 | 0.00% | 66 |
|  | Statistics with study removed | 5.65 | 3.99–7.31 | <0.00001 | 36.43% [127] | – |
|  |  | 5.33 | 4.43–6.24 | <0.00001 | -25.56% [87] | – |
| LVSP [mmHg] (D, difference in means) | Overall effect | 2.46 | -5.25–10.17 | P>0.05 | 0.00% | 57 |
|  | Statistics with study removed | 2.51 | -6.15–11.17 | P>0.05 | 12.33% [125] | – |
|  |  | -0.29 | -6.45–5.87 | P>0.05 | -20.08%....[128] | – |
| LVH  (R, response ratio) | Overall effect | 1.21 | 1.16–1.26 | <0.00001 | 0.00% | 109 |
|  | Statistics with study removed | 1.22 | 1.16–1.27 | <0.00001 | 5.99% [79] | – |
|  |  | 1.20 | 1.16–1.25 | <0.00001 | -8.08 [133] | – |
| d*P*/d*t*_max_ [mmHg/sec]  (D, difference in means) | Overall effect | -1391.05 | -2241.99–(-540.10) | P=0.001 | 0.00% | 38 |
|  | Statistics with study removed | -1383.55 | -2327.66–(-439.44) | P=0.004 | 10.95 [87] | – |
|  |  | -1573.19 | -2175.99–(970.39) | <0.00001 | -29.16%...[112] | – |
| d*P*/d*t*_min_ [mmHg/sec]  (D, difference in means) | Overall effect | -1463.61 | -1876.44–(1050.79) | <0.00001 | 0.00% | 33 |
|  | Statistics with study removed | -1499.98 | -1947.61–(1052.34) | <0.00001 | 8.43% [112] | – |
|  |  | -1393.42 | -1788.32–(-998.53) | <0.00001 | -4.34% [110] | – |
| BW [g] (D, difference in means) | Overall effect | -18.90 | -20.73–(-17.13) | <0.00001 | 0.00% | 93 |
|  | Statistics with study removed | -19.99 | -21.97–(-18.02) | <0.00001 | 10.04% [46] | – |
|  |  | -17.39 | -19.09–(-15.66) | <0.00001 | -5.36% [94] | – |
| Lung mass  (R, response ratio) | Overall effect | 1.56 | 1.47–1.66 | <0.00001 | 0.00% | 49 |
|  | Statistics with study removed | 1.57 | 1.47–1.68 | <0.00001 | 12.87% [24] | – |
|  |  | 1.55 | 1.47–1.64 | <0.00001 | -10.08% [69] | – |
| LV fibrosis  (R, response ratio) | Overall effect | 2.33 | 1.78–3.04 | <0.00001 | 0.00% | 17 |
|  | Statistics with study removed | 2.41 | 1.79–3.25 | <0.00001 | 15.84% [65] | – |
|  |  | 2.08 | 1.62–2.67 | <0.00001 | -17.23% [56] | – |
| PA remodeling  (R, response ratio) | Overall effect | 1.68 | 1.53–1.84 | <0.00001 | 0.00% | 42 |
|  | Statistics with study removed | 1.69 | 1.53–1.87 | <0.00001 | 10.54% [116] | – |
|  |  | 1.65 | 1.51–1.80 | <0.00001 | -6.69% [40] | – |

BW – body weight; d*P*/d*t*_max_ – maximal rate of pressure rise [mmHg/sec]; d*P*/d*t*_min_ –maximal rate of pressure fall [mmHg/sec]; EF – ejection fraction [%]; LV – left ventricle; LVEDd – left ventricular end-diastolic dimension; LVEDP − left ventricular end-diastolic pressure; LVESd – left ventricular end-systolic dimension; LVH – left ventricular hypertrophy; LVSP – left ventricular systolic pressure; mPAP – mean pulmonary artery pressure; RVSP – right ventricular systolic pressure; RVH – right ventricular hypertrophy.* – upper and lower limit; ** – In case where the results of more than one comparison: Sham vs. Vehicle, were reported in single study.

| **Author** | **Year** | **Species**  **Supplementary Table 3.** Study characteristics | **Strain** | **Sex** | **Age (weeks)** | **Animal model** | **Intervention** | **Comparator** | **Duration of experiment (wks)** |
| --- | --- | --- | --- | --- | --- | --- | --- | --- | --- |
| Agrawal V | 2018 | mouse | AKR  CH3 | male | 8 | HFD | 60%kcal fat | 15%kcal fat diet | 20 |
| Ahn BH | 2003 | rat | Sprague-Dawley (SD) | male | 13 | MCT | 60 mg (single inj.) | placebo | 4 |
| Akazawa Y | 2020 | rat | Sprague-Dawley (SD) | male | 6 | SU5416+chronic hypoxia+normoxia  PAB  MCT | 20 mg+10%O_2_  pulmonary artery banding/constriction  60 mg (single inj.) | placebo+21%O_2_  sham-operated  placebo | 6  5  5 |
| Akhavein F | 2007 | rat | Sprague-Dawley (SD) | male |  | MCT | 50 mg (single inj.) | placebo | 3 |
| Alencar AK | 2017 | rat | Wistar | male |  | MCT | 60 mg (single inj.) | placebo | 4 |
| Alencar AKN | 2018 | rat | Wistar | female | 9 | MCT | 60 mg (single inj.) | placebo | 4 |
| Andersen S | 2019 | rat | Sprague-Dawley (SD)  Wistar | male |  | SU5416+chronic hypoxia  PTB | 20 mg+10%O_2_  pulmonary trunk banding | placebo+21%O_2_  sham-operated | 6 |
| Ben Driss A | 2017 | rat | Wistar | male |  | MI | ligation of left coronary artery | sham-operated | 16 |
| Borgdorff MA | 2013 | rat | Wistar | male |  | PAB | pulmonary artery banding/constriction | sham-operated | 11 |
| Campen MJ | 2005 | mouse | C57BL/6J | male | 10-18 | chronic hypoxia | 10%O_2_ | 21%O_2_ | 5 |
| Chabot A | 2011 | rat | Wistar | male |  | MI | ligation of left coronary artery | sham-operated | 5 |
| Chen IC | 2017 | rat | Wistar |  | 6 | AB | ascending aorta banding | sham-operated | 6 |
| Chen IC | 2020 | rat | Wistar | male | 6 | AB | ascending aorta banding | sham-operated | 6 |
| Chen JJ | 2019 | mouse | C57BL/6 |  | 10 | TAC | transverse aortic constriction | sham-operated | 8 |
| Chen Y | 2011 | mouse | C57B6J | male | 10-13 | chronic hypoxia  TAC (M-HF)  TAC (HF) | 10%O_2_  transverse aortic constriction  transverse aortic constriction | 21%O_2_  sham-operated  sham-operated | 3 |
| Chou SH | 2006 | rat | Wistar | male |  | AB | aorta banding | sham-operated | 4  7 |
| Clements RT | 2019 | rat | Sprague-Dawley (SD) | male |  | SU5416+chronic hypoxia+normoxia | 20mg+10%O_2_ | placebo+21%O_2_ |  |
| Correia-Pinto J | 2009 | rat | Wistar | male | 7 | MCT | 60 mg (single inj.) | placebo | 4  6 |
| Dai ZK | 2004 | rat | Wistar | male | 6 | AB | aorta banding | sham-operated | 12 |
| Dai ZK | 2010 | rat | Wistar |  | 6 | AB | aorta banding | sham-operated | 4 |
| Dai ZK | 2011 | rat | Wistar | male | 6 | AB | aorta banding | sham-operated | 4  6 |
| Dayeh NR | 2018 | mouse | C57BL/6 | both | 8-12 | MI | ligation of left coronary artery | sham-operated | 4 |
| de Jong JW | 2000 | rat | Sprague-Dawley (SD) | male |  | MCT | 50 mg (single inj.) | placebo | 4 |
| Deuchar GA | 2002 | rabbit | New Zealand White | male |  | MI | ligation of left coronary artery | sham-operated | 8 |
| Enache I | 2013 | rat | Wistar | male | adult | MCT | 60 mg (single inj.) | placebo | 2  4 |
| Fowler ED | 2018 | rat | Wistar | male |  | MCT | 60 mg (single inj.) | placebo | 4 |
| Fowler ED | 2019 | rat | Wistar | male |  | MCT | 60 mg (single inj.) | placebo | 2 |
| Frede W | 2020 | rat | Sprague-Dawley (SD) | male | 14-15 | MCT | 60 mg (single inj.) | placebo | 3 |
| Fujimori A | 2004 | rat | Sprague-Dawley (SD) | male | 5-6 | MI  MCT | ligation of left coronary artery 60 mg (single inj.) | sham-operated  placebo | 30  4 |
| Fujimoto Y | 2017 | rat | Sprague-Dawley (SD) |  | 5 | LAS | left atrial stenosis | sham-operated | 10 |
| Gomez O | 2018 | rat | Sprague-Dawley (SD) | male | 6 | MCT | 60 mg (single inj.) | placebo | 3 |
| Gong L | 2020 | mouse | CD1 | male | 8 | PAB  TAC | pulmonary artery banding/constriction  transverse aortic constriction | sham-operated | 4  8 |
| Han JC | 2018 | rat | Wistar | male | 9-10 | MCT | 60 mg (single inj.) | placebo | 5 |
| Hansmann G | 2007 | mouse | C57Bl/7 | male | 4 | ApoE-/-+HFD | ApoE-/-+60%kcal fat  ApoE-/- | wild-type+60%kcal fat  wild-type | 10  4 |
| Hardziyenka M | 2011 | rat | Wistar | male | 8 | MCT | 60 mg (single inj.) | placebo | 1 |
| Henriques-Coelho T | 2004 | rat | Wistar | male |  | MCT | 50 mg (single inj.) | placebo | 3 |
| Hentschel T | 2007 | rat | Sprague-Dawley (SD) | male | juvenile | SAB | supracoronary aorta banding | sham-operated | 9 |
| Hessel MH | 2006 | rat | Wistar | male |  | MCT | 30 mg (single inj.)  60 mg (single inj.) | placebo | 4 |
| Hillestad V | 2015 | mouse | C57Bl/6 | male | 6 | chronic hypoxia | 10%O_2_ | 21%O_2_ | 2 |
| Hiram R | 2019 | rat | Wistar | male |  | MCT | 60 mg (single inj.) | placebo | 3 |
| Hoffmann J | 2010 | rat | Sprague-Dawley (SD) |  |  | SAB  MCT | supracoronary aorta banding  60 mg (single inj.) | sham-operated  placebo | 9  3 |
| Hołda MK | 2018 | rat | Wistar | male | 6 | MCT | 60 mg (single inj.) | placebo | 3  6*  6** |
| Hsieh PC | 2006 | rat | Sprague-Dawley (SD) | male |  | MI | ligation of left coronary artery | sham-operated | 16 |
| Huang W | 2021 | rat | Sprague-Dawley (SD) | male | 3-4 | PAB | mechanical stretching of pulmonary vein | sham-operated | 25 days |
| Hunt JM | 2013 | rat | Sprague-Dawley (SD) | male | 2-3 | AB | aorta banding | sham-operated | 9 |
| Ikeda S | 2014 | mouse |  | male |  | PAB  TAC | pulmonary artery banding/constriction  transverse aortic constriction | sham-operated | 4 |
| Imai Y | 2018 | mouse | C57BL/6J | male | 7-10 | TAC | transverse aortic constriction | sham-operated | 2 days |
| Ishikawa K | 1995 | rat | Wistar | male |  | MCT | 60 mg (single inj.) | placebo | 4 |
| Ishikawa S | 1992 | rat | Sprague-Dawley (SD) | male |  | MCT | 60 mg (single inj.) | placebo | 4 |
| Itoh A | 2009 | rat | Wistar | male | 8 | chronic hypoxia | 10%O_2_ | 21%O_2_ | 8 |
| Jasmin JF | 2003 | rat | Wistar | male |  | MI | ligation of left coronary artery | sham-operated | 2  8 |
| Jasmin JF | 2004 | rat | Wistar | male |  | MI | ligation of left coronary artery | sham-operated | 2 |
| Jayasekera G | 2020 | rat | Sprague-Dawley (SD) | male | 3 | SU5416+chronic hypoxia+normoxia | 20mg+10%O_2_ | placebo+21%O_2_ | 5  8 |
| Jeffery TK | 1999 | rat | Wistar | male | 8-9 | chronic hypoxia | 10%O_2_ | 21%O_2_ | 4 |
| Jiang BH | 2010 | rat |  |  |  | MI | ligation of left coronary artery | sham-operated | 5 |
| Jiang BH | 2011 | rat | Charles River | male |  | MI | ligation of left coronary artery | sham-operated | 5 |
| Jiang BH | 2011 | rat |  |  |  | MI | ligation of left coronary artery | sham-operated | 5 |
| Joki Y | 2022 | mouse | C57BL/6J | male | 8 | TAC  HFD | transverse aortic constriction  60%kcal fat | sham-operated  15%kcal fat diet | 5  20 |
| Kelly NJ | 2020 | mouse | C57BL/6J  AKR/J  BALB/cByJ  C57L/J  FVB/NJ  BALB/cJ | male | 6-12 | HFD | 60%kcal fat | 15%kcal fat diet | 20 |
| Kheyfets VO | 2020 | mouse | C57Bl/6 | male | 10-14 | PAB | pulmonary artery banding/constriction | sham-operated | 7 |
| Kingsbury MP | 2003 | guinea pig | Dunkin-Hartley | male |  | AB | ascending aorta banding | sham-operated | 22 |
| Kowalski J | 2021 | mouse | C57BL/6 | male | 8-16 | chronic hypoxia | 10%O_2_ | 21%O_2_ | 6 |
| Lai YC | 2016 | rat | ZSF1 | male | 8-20 | ZSF1 Obese+SU5416 | ZSF1 obese+100 mg | ZSF1 lean+placebo | 14 |
| Lambert M | 2020 | rat |  |  |  | AB  Kcnk3-mut  AB+Kcnk3-mut | ascending aorta banding  Kcnk3-mut  ascending aorta banding  +Kcnk3-mut | sham-operated  wild-type  sham-operated+wild-type | 3 |
| Lamberts RR | 2007 | rat | Wistar | male | 6 | MCT | 30 mg (single inj.) | placebo | 4 |
| Lamberts RR | 2007 | rat | Wistar | male |  | MCT | 60 mg (single inj.) | placebo | 4 |
| Lefebvre F | 2006 | rat | Wistar | male |  | MCT | 60 mg (single inj.) | placebo | 2 |
| Leichsenring-Silva F | 2011 | rat | Wistar | male |  | MCT | 60 mg (single inj.) | placebo | 1  3  31 days |
| Leineweber K | 2000 | rat | Wistar | male | 6 | MCT | 60 mg (single inj.) | placebo | 6 |
| Leineweber K | 2002 | rat | Wistar | male | 6 | MCT | 50 mg (single inj.) | placebo | 4 |
| Li H | 2021 | rat | Sprague-Dawley (SD) | male |  | MCT  PTB | 60 mg (single inj.)  pulmonary trunk banding | placebo  sham-operated | 5  4 |
| Liu Q | 2018 | rat | Wistar | male |  | TAC | transverse aortic constriction | sham-operated | 9 |
| López Y | 2021 | rat | Sprague-Dawley (SD) | male | 8 | STZ | 60 mg (single inj.) | placebo | 4  8  12 |
| Lourenço AP | 2011 | rat | Wistar | male | 7 | MCT  MCT+HFD | 60 mg (single inj.)  placebo+35%kcal fat diet | placebo  placebo+15%kcal fat diet | 7 |
| Lu Y | 2017 | mouse  rat | C57BL/6  Sprague-Dawley (SD) | male |  | chronic hypoxia  MCT | 10%O_2_  60 mg (single inj.) | 21%O_2_  placebo | 3  4 |
| Lu Y | 2017 | rat  mouse | Sprague-Dawley (SD)  C57BL/6 | male |  | MCT  chronic hypoxia | 40 mg (single inj.)  10.5%O_2_ | placebo  21%O_2_ | 3 |
| Lucas M | 2001 | rat | Wistar | male | 7-8 | MI | ligation of left coronary artery | sham-operated | 4 |
| Mathew R | 2000 | rat | Wistar-Kyoto (WKY) | male | 8 | SHRSP | SHRSP | Wistar-Kyoto (WKY) | 7 |
| McNair BD | 2021 | mouse | C57Bl6 | male | 72 | chronic hypoxia | 10%O_2_ | 21%O_2_ | 8 |
| Meng Q | 2017 | mouse | AKR/J | male | 6-12 | HFD | 60%kcal fat | 15%kcal fat diet | 16 |
| Menon DP | 2021 | mouse |  | both | 10-12 | SU5416+chronic hypoxia | 20 mg+10%O_2_ | placebo+21%O_2_ | 4 |
| Minami S | 2004 | rat | Sprague-Dawley (SD) | male |  | MCT | 40 mg (single inj.) | placebo | 2  5  7 |
| Morii I | 1997 | rat | Dahl-Iwai | male | 6 | Dahl salt-sensitive | 8% NaCl | 0.3% NaCl | 12 |
| Morrell NW | 1997 | rat | Sprague-Dawley (SD) | male |  | chronic hypoxia | 10%O_2_ | 21%O_2_ | 1  2 |
| Nakai G | 2019 | mouse | C57BL6 | male | 6 | MCT | 300 mg (twice a week) | placebo | 3  6 |
| Nakata TM | 2015 | rat | Wistar | male | 12 | MCT | 60 mg (single inj.) | placebo | 4 |
| Nguyen QT | 1998 | rat | Wistar | male | 7 | MI | ligation of left coronary artery | sham-operated | 4 |
| Nguyen QT | 2000 | rat | Wistar | male | 7 | MI | ligation of left coronary artery | sham-operated | 4 |
| Nguyen QT | 2020 | rat | Wistar | male |  | MI | ligation of left coronary artery | sham-operated | 2 |
| Nielsen EA | 2017 | rat  rabbit | Sprague-Dawley (SD)  New Zealand White | male | 6 | MCT  PAB | 60 mg (single inj.)  pulmonary artery banding | placebo  sham-operated | 5  3 |
| Okada M | 2009 | rat | Wistar | male | 6 | MCT | 60 mg (single inj.) | placebo | 24 days |
| Okumura K | 2015 | rat | Sprague-Dawley (SD) | male | 6 | MCT | 60 mg (single inj.) | placebo | 3 |
| Pfeifer M | 1998 | rat | Wistar | male |  | AB | ascending aorta banding | sham-operated | 12 |
| Pham T | 2018 | rat | Wistar | male |  | MCT | 60 mg (single inj.) | placebo | 4 |
| Philip JL | 2019 | mouse | C57/Bl6 | male | 6 | MI | ligation of left coronary artery | sham-operated | 12 |
| Piao L | 2012 | rat | Sprague-Dawley (SD) | male |  | PAB  MCT  SU5416+chronic hypoxia | pulmonary artery banding  60 mg (single inj.)  20 mg+10%O_2_ | sham-operated  placebo  placebo+21%O_2_ | 4 |
| Power AS | 2019 | rat | Wistar | male |  | MCT | 60 mg (single inj.) | placebo | 6 |
| Pradhan K | 2016 | mouse | C57Bl/6N | male | 8-10 | TAC | transverse aortic constriction | sham-operated | 8 |
| Qin T | 2022 | rat | Sprague-Dawley (SD) | male |  | MCT | 60 mg (single inj.) | placebo | 4 |
| Radik M | 2019 | rat | Wistar | male | 12 | MCT | 60 mg (single inj.) | placebo | 1  2  4 |
| Ranchoux B | 2018 | rat | Wistar | male | 6 | SAB  HFD  SAB+HFD | supracoronary aorta banding  60%kcal fat  supracoronary aorta banding+60%kcal fat | sham-operated  15%kcal fat diet  sham-operated+15%kcal fat diet | 9 |
| Ravi Y | 2013 | rat | Sprague-Dawley (SD) | male |  | MI | ligation of left coronary artery | sham-operated | 4 |
| Rawat DK | 2014 | rat | Sprague-Dawley (SD) | male |  | SU5416+chronic hypoxia | 20 mg+10%O_2_ | placebo+21%O_2_ | 3 |
| Sabourin J | 2018 | rat | Wistar | male |  | MCT | 60 mg (single inj.) | placebo | 1  2  3 |
| Sakai S | 1996 | rat | Sprague-Dawley (SD) | male |  | MI | ligation of left coronary artery | sham-operated | 2 |
| Satoh T | 2021 | rat | ZSF1 | male | 8 | ZSF1 Obese+SU5416 | ZSF1 lean+100 mg  ZSF1 obese+100 mg  ZSF1 obese | ZSF1 lean+placebo  ZSF1 lean+placebo  ZSF1 lean | 8 |
| Shang L | 2020 | mouse | Balb/C | male |  | TAC | transverse aortic constriction | sham-operated | 6 |
| Shi ZL | 2018 | mouse | Balb/C | male | 6-8 | TAC | transverse aortic constriction | sham-operated | 4 |
| Shirai M | 2013 | rat |  | male | 14 | Dahl salt-sensitive | 8% NaCl | Dahl salt-resistant | 8 |
| Sia YT | 2002 | rat | Wistar | male |  | MI | ligation of left coronary artery | sham-operated | 100 days |
| Spyropoulos F | 2021 | rat | Sprague-Dawley (SD) | male | 12 | SU5416+chronic hypoxia+normoxia | 20 mg+10%O_2_ | placebo+21%O_2_ | 24 days |
| Sun F | 2018 | rat | Wistar | male | 7 | MCT | 60 mg (single inj.) | placebo | 1  3  6 |
| Tian L | 2020 | rat | Sprague-Dawley (SD) | male | 5 | SAB  MCT  SAB+MCT | supracoronary aorta banding  60 mg (single inj.)  supracoronary aorta banding+60 mg (single inj.) | sham-operated  placebo  sham-operated+placebo | 4 |
| Toldo S | 2011 | mouse | out-bred ICR | male | adult | MI | ligation of left coronary artery | sham-operated | 1 |
| Trammell AW | 2017 | mouse | FVB/N | male | 10-14 | transgenic+STZ  transgenic  HFD  transgenic+HFD | BMPR2^R899X^+150 mg  BMPR2^R899X^  60%kcal fat diet  BMPR2^R899X^+60%kcal fat diet | wild-type+150 mg  wild-type  16%kcal fat diet  wild-type+16%kcal fat diet | 6 |
| Tual L | 2006 | rat | Wistar | male |  | chronic hypoxia | 10%O_2_ | 21%O_2_ | 2 |
| Umar S | 2020 | mouse | LDR-KO | male | 60 | HFD | 60%kcal fat diet | 15%kcal fat diet | 12 |
| Umar S | 2012 | rat | Sprague-Dawley (SD) | male | 9-12 | MCT | 60 mg (single inj.) | placebo | 4 |
| Usui S | 2006 | rat | Wistar | male | 6 | MCT | 60 mg (single inj.) | placebo | 6 |
| Vázquez-Garza E | 2020 | rat | Sprague-Dawley (SD) | male | adult | MCT | 60 mg (single inj.) | placebo | 6 |
| Wang L | 2020 | rat | ZSF1 | male | 8 | ZSF1 Obese+SU5416  HFD | ZSF1 obese+100 mg  60%kcal fat diet | ZSF1 lean+placebo  15%kcal fat diet | 14 |
| Wang Q | 2016 | rat | Sprague-Dawley (SD) | male |  | AB | ascending aorta banding | sham-operated | 9 |
| Wang X | 2017 | rat | Sprague-Dawley (SD) | male | 5-6 | MI | ligation of left coronary artery | sham-operated | 4 |
| Wang Y | 2019 | rat | Wistar |  | 12 | MCT | 50 mg (single inj.) | placebo | 4  6 |
| Wittmer VL | 2015 | rat | Wistar | male | 9 | MCT | 60 mg (single inj.) | placebo | 3 |
| Wu J | 2022 | rat | Sprague-Dawley (SD) | male |  | MCT | 60 mg (single inj.) | placebo | 5 |
| Xiong PY | 2022 | rat | Sprague-Dawley (SD) | male | 5 | LAS | left atrial stenosis | sham-operated | 6 |
| Xiong PY | 2018 | rat | Sprague-Dawley (SD) | male | 5 | SAB | supracoronary aorta banding | sham-operated | 8 |
| Yin J | 2011 | rat | Sprague-Dawley (SD) | male |  | SAB | supracoronary aorta banding | sham-operated | 9 |
| Yin N | 2009 | rat | Sprague-Dawley (SD) | male | adult | SAB | supracoronary aorta banding | sham-operated | 9 |
| Yue W | 2019 | mouse | Balb/C | male | 5 | TAC | transverse aortic constriction | sham-operated | 5 |
| Zaky A | 2021 | rat | Sprague-Dawley (SD) | male | adult | SU5416+chronic hypoxia | 20 mg+10%O_2_ | placebo+21%O_2_ | 3  5 |
| Zhang H | 2020 | rat | Sprague-Dawley (SD) | male | 3-4 | PAB | mechanical stretching of pulmonary vein | sham-operated | 25 days |
| Zhang YT | 2019 | rat | Sprague-Dawley (SD) | male |  | AB | aorta banding | sham-operated | 9 |
| Zhuang R | 2018 | rat | Sprague-Dawley (SD) | male | 3-4 | AB | aorta banding | sham-operated | 4 |

**Supplementary Table 4.** The mean values of left ventricle ejection fraction (LVEF%) according to animal model.

| Model | Mean EF% (95%CI)  in Vehicle group |
| --- | --- |
| Aortic banding (AB) | 74.59 (72.86 ‒76.33) |
| ApoE^-^/^-^ + HFD | 77.5 (72.77 – 82.23) |
| Chronic hypoxia (CH) | 58.79 (55.00 ‒62.58) |
| High-fat diets (HFD) | 67.37 (60.87 ‒ 73.89) |
| Left atrial stenosis (LAS) | 64.90 (60.52 – 69.28) |
| Ligation of left coronary artery (MI) | 42.52 (32.79 – 52.25) |
| Monocrotaline inj. (MCT) | 63.82 (55.78 ‒ 71.86) |
| Monocrotaline inj. (MCT) + HFD | 57.90 (54.94 – 60.86) |
| Pulmonary artery banding (PAB)/pulmonary artery constriction (PAC)/pulmonary trunk banding (PTB) | 70.32 (54.67 ‒85.98) |
| SU5416+chronic hypoxia (+normoxia) | 67.18 (59.73 ‒ 74.64) |
| Supracoronary aortic banding (SAB) | 81.41 (74.55 ‒ 88.27) |
| Supracoronary aortic banding (SAB) + HFD | 85.40 (83.65 – 87.15) |
| Transverse aortic constriction (TAC) | 40.83 (26.57 ‒ 55.09) |
| ZSF1 Obese | 67.02 (64.82 ‒ 69.22) |
| ZSF1 Obese + SU5416 | 68.69 (65.93 ‒ 71.46) |

**Supplementary Table 5.** The alterations in body weight (BW) according to animal model.

| Model | Species | BW* (D, difference in means) (95%CI) [g] | P value |
| --- | --- | --- | --- |
| Aortic banding (AB) | Rat | -75.73 (-115.06 ‒(-36.40)) | P<0.0001 |
| Chronic hypoxia (CH) | Rat | -51.49 (-70.01 – 30.97) | P<0.0001 |
|  | Mouse | -1.58 (-3.22 – 0.06) | P=0.05 |
| High-fat diets (HFD) | Mouse | 11.00 (8.44 – 13.56) | P<0.0001 |
|  | Rat | 14.00 (9.81 – 18.19) | P<0.0001 |
| Ligation of left coronary artery (MI) | Mouse | -0.20 (-0.63 – 0.23) | NS |
|  | Rat | -15.01 (-27.37 – (-2.65)) | P=0.017 |
| Monocrotaline inj. (MCT) | Rat | -43.76 (-54.04 – (33.47) | P<0.0001 |
| Monocrotaline inj. (MCT) + HFD | Rat | -18.00 (-21.70 – (-14.30)) | P<0.0001 |
| Pulmonary artery banding (PAB)/pulmonary artery constriction (PAC)/pulmonary trunk banding (PTB) | Mouse | -1.30 (-1.59 – (-1.01)) | P<0.0001 |
|  | Rat | -32.14 (-46.01 – (-18.26) | P<0.0001 |
| SU5416+chronic hypoxia (+normoxia) | Rat | 31.00 (-27.35 – 89.34) | NS |
| Supracoronary aortic banding (SAB) | Rat | -10.00 (-22.39 – 2.39) | NS |
| Transverse aortic constriction (TAC) | Mouse | -1.16 (-2.52 – 0.20) | NS |
|  | Rat | 29.14 (7.53 – 50.47) | P<0.0001 |
| ZSF1 Obese + SU5416 | Rat | 140 (22.4 ‒ 257.6) | P=0.019 |

* at the end of the study

**Supplementary Material .** Study references

1. Agrawal V, Fortune N, Yu S, Fuentes J, Shi F, Nichols D, Gleaves L, Poovey E, Wang TJ, Brittain EL, Collins S, West JD, Hemnes AR. Natriuretic peptide receptor C contributes to disproportionate right ventricular hypertrophy in a rodent model of obesity-induced heart failure with preserved ejection fraction with pulmonary hypertension. Pulm Circ. 2019 Dec 18;9(4):2045894019878599. doi: 10.1177/2045894019895452
2. Ahn BH, Park HK, Cho HG, Lee HA, Lee YM, Yang EK, Lee WJ. Estrogen and enalapril attenuate the development of right ventricular hypertrophy induced by monocrotaline in ovariectomized rats. J Korean Med Sci. 2003 Oct;18(5):641-8. doi: 10.3346/jkms.2003.18.5.641.
3. Akazawa Y, Okumura K, Ishii R, Slorach C, Hui W, Ide H, Honjo O, Sun M, Kabir G, Connelly K, Friedberg MK. Pulmonary artery banding is a relevant model to study the right ventricular remodeling and dysfunction that occurs in pulmonary arterial hypertension. J Appl Physiol (1985). 2020 Aug 1;129(2):238-246. doi: 10.1152/japplphysiol.00148.2020.
4. Akhavein F, St-Michel EJ, Seifert E, Rohlicek CV. Decreased left ventricular function, myocarditis, and coronary arteriolar medial thickening following monocrotaline administration in adult rats. J Appl Physiol (1985). 2007 Jul;103(1):287-95. doi: 10.1152/japplphysiol.01509.2005.
5. Alencar AK, Montes GC, Montagnoli T, Silva AM, Martinez ST, Fraga AG, Wang H, Groban L, Sudo RT, Zapata-Sudo G. Activation of GPER ameliorates experimental pulmonary hypertension in male rats. Eur J Pharm Sci. 2017 Jan 15;97:208-217. doi: 10.1016/j.ejps.2016.11.009.
6. Alencar AKN, Montes GC, Costa DG, Mendes LVP, Silva AMS, Martinez ST, Trachez MM, Cunha VDMN, Montagnoli TL, Fraga AGM, Wang H, Groban L, Fraga CAM, Sudo RT, Zapata-Sudo G. Cardioprotection Induced by Activation of GPER in Ovariectomized Rats With Pulmonary Hypertension. J Gerontol A Biol Sci Med Sci. 2018 Aug 10;73(9):1158-1166. doi: 10.1093/gerona/gly068.
7. Andersen S, Axelsen JB, Ringgaard S, Nyengaard JR, Hyldebrandt JA, Bogaard HJ, de Man FS, Nielsen-Kudsk JE, Andersen A. Effects of combined angiotensin II receptor antagonism and neprilysin inhibition in experimental pulmonary hypertension and right ventricular failure. Int J Cardiol. 2019 Oct 15;293:203-210. doi: 10.1016/j.ijcard.2019.06.065.
8. Ben Driss A, Devaux C, Henrion D, Duriez M, Thuillez C, Levy BI, Michel JB. Hemodynamic stresses induce endothelial dysfunction and remodeling of pulmonary artery in experimental compensated heart failure. Circulation. 2000 Jun 13;101(23):2764-70. doi: 10.1161/01.cir.101.23.2764.
9. Borgdorff MA, Bartelds B, Dickinson MG, Steendijk P, Berger RM. A cornerstone of heart failure treatment is not effective in experimental right ventricular failure. Int J Cardiol. 2013 Nov 5;169(3):183-9. doi: 10.1016/j.ijcard.2013.08.102.
10. Campen MJ, Shimoda LA, O'Donnell CP. Acute and chronic cardiovascular effects of intermittent hypoxia in C57BL/6J mice. J Appl Physiol (1985). 2005 Nov;99(5):2028-35. doi: 10.1152/japplphysiol.00411.2005.
11. Chabot A, Jiang BH, Shi Y, Tardif JC, Dupuis J. Role of aldosterone on lung structural remodelling and right ventricular function in congestive heart failure. BMC Cardiovasc Disord. 2011 Dec 2;11:72. doi: 10.1186/1471-2261-11-72.
12. Chen IC, Lin JY, Liu YC, Chai CY, Yeh JL, Hsu JH, Wu BN, Dai ZK. The beneficial effects of angiotensin-converting enzyme II (ACE2) activator in pulmonary hypertension secondary to left ventricular dysfunction. Int J Med Sci. 2020 Sep 16;17(16):2594-2602. doi: 10.7150/ijms.48096.
13. Chen IC, Tan MS, Wu BN, Chai CY, Yeh JL, Chou SH, Chen IJ, Dai ZK. Statins ameliorate pulmonary hypertension secondary to left ventricular dysfunction through the Rho-kinase pathway and NADPH oxidase. Pediatr Pulmonol. 2017 Apr;52(4):443-457. doi: 10.1002/ppul.23610.
14. Chen Y, Guo H, Xu D, Xu X, Wang H, Hu X, Lu Z, Kwak D, Xu Y, Gunther R, Huo Y, Weir EK. Left ventricular failure produces profound lung remodeling and pulmonary hypertension in mice: heart failure causes severe lung disease. Hypertension. 2012 Jun;59(6):1170-8. doi: 10.1161/HYPERTENSIONAHA.111.186072.
15. Chou SH, Chai CY, Wu JR, Tan MS, Chiu CC, Chen IJ, Jeng AY, Chang CI, Kwan AL, Dai ZK. The effects of debanding on the lung expression of ET-1, eNOS, and cGMP in rats with left ventricular pressure overload. Exp Biol Med (Maywood). 2006 Jun;231(6):954-9.
16. Chou SH, Chai CY, Wu JR, Tan MS, Chiu CC, Chen IJ, Jeng AY, Chang CI, Kwan AL, Dai ZK. The effects of debanding on the lung expression of ET-1, eNOS, and cGMP in rats with left ventricular pressure overload. Exp Biol Med (Maywood). 2006 Jun;231(6):954-9.
17. Clements RT, Vang A, Fernandez-Nicolas A, Kue NR, Mancini TJ, Morrison AR, Mallem K, McCullough DJ, Choudhary G. Treatment of Pulmonary Hypertension With Angiotensin II Receptor Blocker and Neprilysin Inhibitor Sacubitril/Valsartan. Circ Heart Fail. 2019 Nov;12(11):e005819. doi: 10.1161/CIRCHEARTFAILURE.119.005819.
18. Correia-Pinto J, Henriques-Coelho T, Roncon-Albuquerque R Jr, Lourenço AP, Melo-Rocha G, Vasques-Nóvoa F, Gillebert TC, Leite-Moreira AF. Time course and mechanisms of left ventricular systolic and diastolic dysfunction in monocrotaline-induced pulmonary hypertension. Basic Res Cardiol. 2009 Sep;104(5):535-45. doi: 10.1007/s00395-009-0017-3.
19. Dai ZK, Hsieh CC, Chai CY, Wu JR, Jeng AY, Chou SH, Wu BN, Yeh JL, Chen IJ, Tan MS. Protective effects of a dual endothelin converting enzyme/neutral endopeptidase inhibitor on the development of pulmonary hypertension secondary to cardiac dysfunction in the rat. Pediatr Pulmonol. 2010 Nov;45(11):1076-85. doi: 10.1002/ppul.21290.
20. Dai ZK, Tan MS, Chai CY, Yeh JL, Chou SH, Chiu CC, Jeng AY, Chen IJ, Wu JR. Upregulation of endothelial nitric oxide synthase and endothelin-1 in pulmonary hypertension secondary to heart failure in aorta-banded rats. Pediatr Pulmonol. 2004 Mar;37(3):249-56. doi: 10.1002/ppul.10413.
21. Dai ZK, Wu BN, Chen IC, Chai CY, Wu JR, Chou SH, Yeh JL, Chen IJ, Tan MS. Attenuation of pulmonary hypertension secondary to left ventricular dysfunction in the rat by Rho-kinase inhibitor fasudil. Pediatr Pulmonol. 2011 Jan;46(1):45-59. doi: 10.1002/ppul.21323.
22. Dayeh NR, Tardif JC, Shi Y, Tanguay M, Ledoux J, Dupuis J. Echocardiographic validation of pulmonary hypertension due to heart failure with reduced ejection fraction in mice. Sci Rep. 2018 Jan 22;8(1):1363. doi: 10.1038/s41598-018-19625-2.
23. de Jong JW, Schoemaker RG, de Jonge R, Bernocchi P, Keijzer E, Harrison R, Sharma HS, Ceconi C. Enhanced expression and activity of xanthine oxidoreductase in the failing heart. J Mol Cell Cardiol. 2000 Nov;32(11):2083-9. doi: 10.1006/jmcc.2000.1240.
24. Deuchar GA, Docherty A, MacLean MR, Hicks MN. Pulmonary hypertension secondary to left ventricular dysfunction: the role of nitric oxide and endothelin-1 in the control of pulmonary vascular tone. Br J Pharmacol. 2002 Feb;135(4):1060-8. doi: 10.1038/sj.bjp.0704529.
25. Enache I, Charles AL, Bouitbir J, Favret F, Zoll J, Metzger D, Oswald-Mammosser M, Geny B, Charloux A. Skeletal muscle mitochondrial dysfunction precedes right ventricular impairment in experimental pulmonary hypertension. Mol Cell Biochem. 2013 Jan;373(1-2):161-70. doi: 10.1007/s11010-012-1485-6.
26. Fowler ED, Drinkhill MJ, Norman R, Pervolaraki E, Stones R, Steer E, Benoist D, Steele DS, Calaghan SC, White E. Beta1-adrenoceptor antagonist, metoprolol attenuates cardiac myocyte Ca2+ handling dysfunction in rats with pulmonary artery hypertension. J Mol Cell Cardiol. 2018 Jul;120:74-83. doi: 10.1016/j.yjmcc.2018.05.015.
27. Fowler ED, Hauton D, Boyle J, Egginton S, Steele DS, White E. Energy Metabolism in the Failing Right Ventricle: Limitations of Oxygen Delivery and the Creatine Kinase System. Int J Mol Sci. 2019 Apr 12;20(8):1805. doi: 10.3390/ijms20081805.
28. Frede W, Medert R, Poth T, Gorenflo M, Vennekens R, Freichel M, Uhl S. TRPM4 Modulates Right Ventricular Remodeling Under Pressure Load Accompanied With Decreased Expression Level. J Card Fail. 2020 Jul;26(7):599-609. doi: 10.1016/j.cardfail.2020.02.006.
29. Fujimori A, Miyauchi T, Sakai S, Yuyama H, Iemitsu M, Sanagi M, Sudoh K, Goto K, Shikama H, Yamaguchi I. YM598, an orally active ET(A) receptor antagonist, ameliorates the progression of cardiopulmonary changes and both-side heart failure in rats with cor pulmonale and myocardial infarction. J Cardiovasc Pharmacol. 2004 Nov;44 Suppl doi: 10.1097/01.fjc.0000166304.40937.0b.
30. Fujimoto Y, Urashima T, Kawachi F, Akaike T, Kusakari Y, Ida H, Minamisawa S. Pulmonary hypertension due to left heart disease causes intrapulmonary venous arterialization in rats. J Thorac Cardiovasc Surg. 2017 Nov;154(5):1742-1753.e8. doi: 10.1016/j.jtcvs.2017.06.053.
31. Gomez O, Okumura K, Honjo O, Sun M, Ishii R, Bijnens B, Friedberg MK. Heart rate reduction improves biventricular function and interactions in experimental pulmonary hypertension. Am J Physiol Heart Circ Physiol. 2018 Mar 1;314(3):H542-H551. doi: 10.1152/ajpheart.00493.2017.
32. Gong L, Wang S, Shen L, Liu C, Shenouda M, Li B, Liu X, Shaw JA, Wineman AL, Yang Y, Xiong D, Eichmann A, Evans SM, Weiss SJ, Si MS. SLIT3 deficiency attenuates pressure overload-induced cardiac fibrosis and remodeling. JCI Insight. 2020 Jun 18;5(12):e136852. doi: 10.1172/jci.insight.136852.
33. Han JC, Guild SJ, Pham T, Nisbet L, Tran K, Taberner AJ, Loiselle DS. Left-Ventricular Energetics in Pulmonary Arterial Hypertension-Induced Right-Ventricular Hypertrophic Failure. Front Physiol. 2018 Jan 9;8:1115. doi: 10.3389/fphys.2017.01115.
34. Hansmann G, Wagner RA, Schellong S, Perez VA, Urashima T, Wang L, Sheikh AY, Suen RS, Stewart DJ, Rabinovitch M. Pulmonary arterial hypertension is linked to insulin resistance and reversed by peroxisome proliferator-activated receptor-gamma activation. Circulation. 2007; 115: 1275-84.
35. Hardziyenka M, Campian ME, Reesink HJ, Surie S, Bouma BJ, Groenink M, Klemens CA, Beekman L, Remme CA, Bresser P, Tan HL. Right ventricular failure following chronic pressure overload is associated with reduction in left ventricular mass: evidence for atrophic remodeling. J Am Coll Cardiol. 2011 Feb 22;57(8):921-8. doi: 10.1016/j.jacc.2010.08.648.
36. Henriques-Coelho T, Correia-Pinto J, Roncon-Albuquerque R Jr, Baptista MJ, Lourenço AP, Oliveira SM, Brandão-Nogueira A, Teles A, Fortunato JM, Leite-Moreira AF. Endogenous production of ghrelin and beneficial effects of its exogenous administration in monocrotaline-induced pulmonary hypertension. Am J Physiol Heart Circ Physiol. 2004 Dec;287(6):H2885-90. doi: 10.1152/ajpheart.01122.2003.
37. Hentschel T, Yin N, Riad A, Habbazettl H, Weimann J, Koster A, Tschope C, Kuppe H, Kuebler WM. Inhalation of the phosphodiesterase-3 inhibitor milrinone attenuates pulmonary hypertension in a rat model of congestive heart failure. Anesthesiology. 2007 Jan;106(1):124-31. doi: 10.1097/00000542-200701000-00021.
38. Hessel MH, Steendijk P, den Adel B, Schutte CI, van der Laarse A. Characterization of right ventricular function after monocrotaline-induced pulmonary hypertension in the intact rat. Am J Physiol Heart Circ Physiol. 2006 Nov;291(5):H2424-30. doi: 10.1152/ajpheart.00369.2006.
39. Hillestad V, Espe EK, Cero F, Larsen KO, Sjaastad I, Nygård S, Skjønsberg OH, Christensen G. IL-18 neutralization during alveolar hypoxia improves left ventricular diastolic function in mice. Acta Physiol (Oxf). 2015 Feb;213(2):492-504. doi: 10.1111/apha.12376.
40. Hiram R, Naud P, Xiong F, Al-U'datt D, Algalarrondo V, Sirois MG, Tanguay JF, Tardif JC, Nattel S. Right Atrial Mechanisms of Atrial Fibrillation in a Rat Model of Right Heart Disease. J Am Coll Cardiol. 2019 Sep 10;74(10):1332-1347. doi: 10.1016/j.jacc.2019.06.066.
41. Hoffmann J, Yin J, Kukucka M, Yin N, Saarikko I, Sterner-Kock A, Fujii H, Leong-Poi H, Kuppe H, Schermuly RT, Kuebler WM. Mast cells promote lung vascular remodelling in pulmonary hypertension. Eur Respir J. 2011 Jun;37(6):1400-10. doi: 10.1183/09031936.00043310.
42. Hołda MK, Szczepanek E, Bielawska J, Palka N, Wojtysiak D, Frączek P, Nowakowski M, Sowińska N, Arent Z, Podolec P, Kopeć G. Changes in heart morphometric parameters over the course of a monocrotaline-induced pulmonary arterial hypertension rat model. J Transl Med. 2020 Jun 30;18(1):262. doi: 10.1186/s12967-020-02440-7.
43. Hsieh PC, MacGillivray C, Gannon J, Cruz FU, Lee RT. Local controlled intramyocardial delivery of platelet-derived growth factor improves postinfarction ventricular function without pulmonary toxicity. Circulation. 2006 Aug 15;114(7):637-44. doi: 10.1161/CIRCULATIONAHA.106.639831.
44. Huang W, Liu H, Pan Y, Yang H, Lin J, Zhang H. Mechanical stretching of the pulmonary vein mediates pulmonary hypertension due to left heart disease by regulating SAC/MAPK pathway and the expression of IL-6 and TNF-α. J Cardiothorac Surg. 2021 May 10;16(1):127. doi: 10.1186/s13019-021-01471-5. Erratum in: J Cardiothorac Surg. 2021 Jun 9;16(1):169.
45. Hunt JM, Bethea B, Liu X, Gandjeva A, Mammen PP, Stacher E, Gandjeva MR, Parish E, Perez M, Smith L, Graham BB, Kuebler WM, Tuder RM. Pulmonary veins in the normal lung and pulmonary hypertension due to left heart disease. Am J Physiol Lung Cell Mol Physiol. 2013 Nov 15;305(10):L725-36. doi: 10.1152/ajplung.00186.2013.
46. Ikeda S, Satoh K, Kikuchi N, Miyata S, Suzuki K, Omura J, Shimizu T, Kobayashi K, Kobayashi K, Fukumoto Y, Sakata Y, Shimokawa H. Crucial role of rho-kinase in pressure overload-induced right ventricular hypertrophy and dysfunction in mice. Arterioscler Thromb Vasc Biol. 2014 Jun;34(6):1260-71. doi: 10.1161/ATVBAHA.114.303320.
47. Imai Y, Kariya T, Iwakiri M, Yamada Y, Takimoto E. Sildenafil ameliorates right ventricular early molecular derangement during left ventricular pressure overload. PLoS One. 2018 Apr 5;13(4):e0195528. doi: 10.1371/journal.pone.0195528.
48. Ishikawa K, Hashimoto H, Mitani S, Toki Y, Okumura K, Ito T. Enalapril improves heart failure induced by monocrotaline without reducing pulmonary hypertension in rats: roles of preserved myocardial creatine kinase and lactate dehydrogenase isoenzymes. Int J Cardiol. 1995 Jan 6;47(3):225-33. doi: 10.1016/0167-5273(94)02198-r.
49. Ishikawa S, Honda M, Yamada S, Goto Y, Morioka S, Ishinaga Y, Murakami Y, Masumura S, Moriyama K. Different biventricular remodelling of myosin and collagen in pulmonary hypertension. Clin Exp Pharmacol Physiol. 1992 Oct;19(10):723-32. doi: 10.1111/j.1440-1681.1992.tb00410.x.
50. Itoh A, Tomita H, Sano S. Doppler echocardiographic assessment of left ventricular diastolic function in chronic hypoxic rats. Acta Med Okayama. 2009 Apr;63(2):87-96. doi: 10.18926/AMO/31832.
51. Jasmin JF, Calderone A, Leung TK, Villeneuve L, Dupuis J. Lung structural remodeling and pulmonary hypertension after myocardial infarction: complete reversal with irbesartan. Cardiovasc Res. 2003 Jun 1;58(3):621-31. doi: 10.1016/s0008-6363(03)00290-6.
52. Jasmin JF, Mercier I, Hnasko R, Cheung MW, Tanowitz HB, Dupuis J, Lisanti MP. Lung remodeling and pulmonary hypertension after myocardial infarction: pathogenic role of reduced caveolin expression. Cardiovasc Res. 2004 Sep 1;63(4):747-55. doi: 10.1016/j.card
53. Jayasekera G, Wilson KS, Buist H, Woodward R, Uckan A, Hughes C, Nilsen M, Church AC, Johnson MK, Gallagher L, Mullin J, MacLean MR, Holmes WM, Peacock AJ, Welsh DJ. Understanding longitudinal biventricular structural and functional changes in a pulmonary hypertension Sugen-hypoxia rat model by cardiac magnetic resonance imaging. Pulm Circ. 2020 Feb 10;10(1):2045894019897513. doi: 10.1177/2045894019897513.
54. Jeffery TK, Wanstall JC. Perindopril, an angiotensin converting enzyme inhibitor, in pulmonary hypertensive rats: comparative effects on pulmonary vascular structure and function. Br J Pharmacol. 1999 Dec;128(7):1407-18. doi: 10.1038/sj.bjp.0702923.
55. Jiang BH, Nguyen QT, Tardif JC, Shi Y, Dupuis J. Single measurement of troponin T for early prediction of infarct size, congestive heart failure, and pulmonary hypertension in an animal model of myocardial infarction. Cardiovasc Pathol. 2011 May-Jun;20(3):e85-9. doi: 10.1016/j.carpath.2010.04.005.
56. Jiang BH, Tardif JC, Sauvageau S, Ducharme A, Shi Y, Martin JG, Dupuis J. Beneficial effects of atorvastatin on lung structural remodeling and function in ischemic heart failure. J Card Fail. 2010 Aug;16(8):679-88. doi: 10.1016/j.cardfail.2010.03.003.
57. Jiang BH, Tardif JC, Shi Y, Dupuis J. Bosentan does not improve pulmonary hypertension and lung remodeling in heart failure. Eur Respir J. 2011 Mar;37(3):578-86. doi: 10.1183/09031936.00053710.
58. Joki Y, Konishi H, Takasu K, Minamino T. Tofogliflozin, a sodium-glucose cotransporter 2 inhibitor, improves pulmonary vascular remodeling due to left heart disease in mice. J Cardiol. 2022 Oct 13:S0914-5087(22)00256-8. doi: 10.1016/j.jjcc.2022.10.003.
59. Kelly NJ, Radder JE, Baust JJ, Burton CL, Lai YC, Potoka KC, Agostini BA, Wood JP, Bachman TN, Vanderpool RR, Dandachi N, Leme AS, Gregory AD, Morris A, Mora AL, Gladwin MT, Shapiro SD. Mouse Genome-Wide Association Study of Preclinical Group II Pulmonary Hypertension Identifies Epidermal Growth Factor Receptor. Am J Respir Cell Mol Biol. 2017 Apr;56(4):488-496. doi: 10.1165/rcmb.2016-0176OC.
60. Kheyfets VO, Dufva MJ, Boehm M, Tian X, Qin X, Tabakh JE, Truong U, Ivy D, Spiekerkoetter E. The left ventricle undergoes biomechanical and gene expression changes in response to increased right ventricular pressure overload. Physiol Rep. 2020 May;8(9):e14347. doi: 10.14814/phy2.14347.
61. Kingsbury MP, Huang W, Donnelly JL, Jackson E, Needham E, Turner MA, Sheridan DJ. Structural remodelling of lungs in chronic heart failure. Basic Res Cardiol. 2003 Sep;98(5):295-303. doi: 10.1007/s00395-003-0419-6.
62. Kowalski J, Deng L, Suennen C, Koca D, Meral D, Bode C, Hein L, Lother A. Eplerenone Improves Pulmonary Vascular Remodeling and Hypertension by Inhibition of the Mineralocorticoid Receptor in Endothelial Cells. Hypertension. 2021 Aug;78(2):456-465. doi: 10.1161/HYPERTENSIONAHA.120.16196.
63. Lai YC, Tabima DM, Dube JJ, Hughan KS, Vanderpool RR, Goncharov DA, St Croix CM, Garcia-Ocaña A, Goncharova EA, Tofovic SP, Mora AL, Gladwin MT. SIRT3-AMP-Activated Protein Kinase Activation by Nitrite and Metformin Improves Hyperglycemia and Normalizes Pulmonary Hypertension Associated With Heart Failure With Preserved Ejection Fraction. Circulation. 2016 Feb 23;133(8):717-31. doi: 10.1161/CIRCULATIONAHA.115.018935.
64. Lambert M, Mendes-Ferreira P, Ghigna MR, LeRibeuz H, Adão R, Boet A, Capuano V, Rucker-Martin C, Brás-Silva C, Quarck R, Domergue V, Vachiéry JL, Humbert M, Perros F, Montani D, Antigny F. Kcnk3 Dysfunction Exaggerates The Development Of Pulmonary Hypertension Induced By Left Ventricular Pressure Overload. Cardiovasc Res. 2021 Jan 23:cvab016. doi: 10.1093/cvr/cvab016.
65. Lamberts RR, Caldenhoven E, Lansink M, Witte G, Vaessen RJ, St Cyr JA, Stienen GJ. Preservation of diastolic function in monocrotaline-induced right ventricular hypertrophy in rats. Am J Physiol Heart Circ Physiol. 2007 Sep;293(3):H1869-76. doi: 10.1152/ajpheart.00294.2007.
66. Lamberts RR, Vaessen RJ, Westerhof N, Stienen GJ. Right ventricular hypertrophy causes impairment of left ventricular diastolic function in the rat. Basic Res Cardiol. 2007 Jan;102(1):19-27. doi: 10.1007/s00395-006-0620-5.
67. Lefebvre F, Préfontaine A, Calderone A, Caron A, Jasmin JF, Villeneuve L, Dupuis J. Modification of the pulmonary renin-angiotensin system and lung structural remodelling in congestive heart failure. Clin Sci (Lond). 2006 Sep;111(3):217-24. doi: 10.1042/CS20060027.
68. Leichsenring-Silva F, Tavares AM, Mosele F, Berger B, Llesuy S, Belló-Klein A. Association of the time course of pulmonary arterial hypertension with changes in oxidative stress in the left ventricle. Clin Exp Pharmacol Physiol. 2011 Dec;38(12):804-10. doi: 10.1111/j.1440-1681.2011.0560
69. Leineweber K, Brandt K, Wludyka B, Beilfuss A, Pönicke K, Heinroth-Hoffmann I, Brodde OE. Ventricular hypertrophy plus neurohumoral activation is necessary to alter the cardiac beta-adrenoceptor system in experimental heart failure. Circ Res. 2002 Nov 29;91(11):1056-62. doi: 10.1161/01.res.0000045088.59360.b7.
70. Leineweber K, Seyfarth T, Brodde OE. Chamber-specific alterations of noradrenaline uptake (uptake(1)) in right ventricles of monocrotaline-treated rats. Br J Pharmacol. 2000 Dec;131(7):1438-44. doi: 10.1038/sj.bjp.0703698.
71. Li H, Zhang Y, Wang S, Yue Y, Liu Q, Huang S, Peng H, Zhang Y, Zeng W, Wu Z. Dapagliflozin has No Protective Effect on Experimental Pulmonary Arterial Hypertension and Pulmonary Trunk Banding Rat Models. Front Pharmacol. 2021 Nov 1;12:756226. doi: 10.3389/fphar.2021.756226.
72. Liu Q, Hu H, Hu T, Han T, Wang A, Huang L, Tan Q, Tan W. STVNa attenuates right ventricle hypertrophy and pulmonary artery remodeling in rats induced by transverse aortic constriction. Biomed Pharmacother. 2018 May;101:371-378. doi: 10.1016/j.biopha.2018.02.078.
73. López Y López G, Tepox Galicia AY, Atonal Flores F, Flores Hernández J, Pérez Vizcaino F, Villa Mancera AE, Miguél GG, Reynoso Palomar A. Echocardiographic follow-up to right ventricular modifications in secondary pulmonary hypertension to diabetes in rats. Clin Exp Hypertens. 2021 Apr 3;43(3):242-253. doi: 10.1080/10641963.2020.1860077.
74. Lourenço AP, Vasques-Nóvoa F, Fontoura D, Brás-Silva C, Roncon-Albuquerque R Jr, Leite-Moreira AF. A Western-type diet attenuates pulmonary hypertension with heart failure and cardiac cachexia in rats. J Nutr. 2011 Nov;141(11):1954-60. doi: 10.3945/jn.111.145763.
75. Lu Y, Guo H, Sun Y, Pan X, Dong J, Gao D, Chen W, Xu Y, Xu D. Valsartan attenuates pulmonary hypertension via suppression of mitogen activated protein kinase signaling and matrix metalloproteinase expression in rodents. Mol Med Rep. 2017 Aug;16(2):1360-1368. doi: 10.3892/mmr.2017.6706.
76. Lu Z, Xu X, Hu X, Zhu G, Zhang P, van Deel ED, French JP, Fassett JT, Oury TD, Bache RJ, Chen Y. Extracellular superoxide dismutase deficiency exacerbates pressure overload-induced left ventricular hypertrophy and dysfunction. Hypertension. 2008 Jan;51(1):19-25. doi: 10.1161/HYPERTENSIONAHA.107.098186.
77. Lucas M, Jasmin JF, Dupuis J. Effect of ET(A) receptor antagonist on pulmonary hypertension and vascular reactivity in rats with congestive heart failure. Pulm Pharmacol Ther. 2001;14(4):307-14. doi: 10.1006/pupt.2001.0298.
78. Mathew R, Fan NY, Yuan N, Chander PN, Gewitz MH, Stier CT Jr. Inhibition of NOS enhances pulmonary vascular changes in stroke-prone spontaneously hypertensive rats. Am J Physiol Lung Cell Mol Physiol. 2000 Jan;278(1):L81-9. doi: 10.1152/ajplung.2000.278.1.L81.
79. McNair BD, Schlatter JA, Cook RF, Yusifova M, Bruns DR. Inhibition of mTOR by rapamycin does not improve hypoxic pulmonary hypertension-induced right heart failure in old mice. Exp Gerontol. 2021 Aug;151:111395. doi: 10.1016/j.exger.2021.111395.
80. Meng Q, Lai YC, Kelly NJ, Bueno M, Baust JJ, Bachman TN, Goncharov D, Vanderpool RR, Radder JE, Hu J, Goncharova E, Morris AM, Mora AL, Shapiro SD, Gladwin MT. Development of a Mouse Model of Metabolic Syndrome, Pulmonary Hypertension, and Heart Failure with Preserved Ejection Fraction. Am J Respir Cell Mol Biol. 2017 Apr;56(4):497-505. doi: 10.1165/rcmb.2016-0177OC.
81. Menon DP, Qi G, Kim SK, Moss ME, Penumatsa KC, Warburton RR, Toksoz D, Wilson J, Hill NS, Jaffe IZ, Preston IR. Vascular cell-specific roles of mineralocorticoid receptors in pulmonary hypertension. Pulm Circ. 2021 Jun 18;11(3):20458940211025240. doi: 10.1177/20458940211025240.
82. Minami S, Onodera T, Okazaki F, Miyazaki H, Ohsawa S, Mochizuki S. Myocyte morphological characteristics differ between the phases of pulmonary hypertension-induced ventricular hypertrophy and failure. Int Heart J. 2006 Jul;47(4):629-37. doi: 10.1536/ihj.47.629.
83. Morii I, Kihara Y, Inoko M, Sasayama S. Myocardial contractile efficiency and oxygen cost of contractility are preserved during transition from compensated hypertrophy to failure in rats with salt-sensitive hypertension. Hypertension. 1998 Apr;31(4):949-60. doi: 10.1161/01.hyp.31.4.949.
84. Morrell NW, Danilov SM, Satyan KB, Morris KG, Stenmark KR. Right ventricular angiotensin converting enzyme activity and expression is increased during hypoxic pulmonary hypertension. Cardiovasc Res. 1997 May;34(2):393-403. doi: 10.1016/s0008-6363(97)00049-7.
85. Nakai G, Shimura D, Uesugi K, Kajimura I, Jiao Q, Kusakari Y, Soga T, Goda N, Minamisawa S. Pyruvate dehydrogenase activation precedes the down-regulation of fatty acid oxidation in monocrotaline-induced myocardial toxicity in mice. Heart Vessels. 2019 Mar;34(3):545-555. doi: 10.1007/s00380-018-1293-3.
86. Nakata TM, Tanaka R, Yoshiyuki R, Fukayama T, Goya S, Fukushima R. Effects of Single Drug and Combined Short-term Administration of Sildenafil, Pimobendan, and Nicorandil on Right Ventricular Function in Rats With Monocrotaline-induced Pulmonary Hypertension. J Cardiovasc Pharmacol. 2015 Jun;65(6):640-8. doi: 10.1097/FJC.0000000000000236.
87. Nguyen QT, Cernacek P, Calderoni A, Stewart DJ, Picard P, Sirois P, White M, Rouleau JL. Endothelin A receptor blockade causes adverse left ventricular remodeling but improves pulmonary artery pressure after infarction in the rat. Circulation. 1998 Nov 24;98(21):2323-30. doi: 10.1161/01.cir.98.21.2323.
88. Nguyen QT, Colombo F, Rouleau JL, Dupuis J, Calderone A. LU135252, an endothelin(A) receptor antagonist did not prevent pulmonary vascular remodelling or lung fibrosis in a rat model of myocardial infarction. Br J Pharmacol. 2000 Aug;130(7):1525-30. doi: 10.1038/sj.bjp.0703466.
89. Nguyen QT, Nsaibia MJ, Sirois MG, Calderone A, Tardif JC, Fen Shi Y, Ruiz M, Daneault C, Gagnon L, Grouix B, Laurin P, Dupuis J. PBI-4050 reduces pulmonary hypertension, lung fibrosis, and right ventricular dysfunction in heart failure. Cardiovasc Res. 2020 Jan 1;116(1):171-182. doi: 10.1093/cvr/cvz034.
90. Nielsen EA, Okumura K, Sun M, Hjortdal VE, Redington AN, Friedberg MK. Regional septal hinge-point injury contributes to adverse biventricular interactions in pulmonary hypertension. Physiol Rep. 2017 Jul;5(14):e13332. doi: 10.14814/phy2.13332.
91. Okada M, Harada T, Kikuzuki R, Yamawaki H, Hara Y. Effects of telmisartan on right ventricular remodeling induced by monocrotaline in rats. J Pharmacol Sci. 2009 Oct;111(2):193-200. doi: 10.1254/jphs.09112fp.
92. Okumura K, Kato H, Honjo O, Breitling S, Kuebler WM, Sun M, Friedberg MK. Carvedilol improves biventricular fibrosis and function in experimental pulmonary hypertension. J Mol Med (Berl). 2015 Jun;93(6):663-74. doi: 10.1007/s00109-015-1251-9.
93. Pfeifer M, Bruckschlegel G, Holmer SR, Paul M, Riegger AJ, Schunkert H. Reciprocal regulation of pulmonary and cardiac angiotensin-converting enzyme in rats with severe left ventricular hypertrophy. Cardiovasc Res. 1998 Apr;38(1):125-32. doi: 10.1016/s0008-6363(97)00298-8.
94. Pham T, Nisbet L, Taberner A, Loiselle D, Han JC. Pulmonary arterial hypertension reduces energy efficiency of right, but not left, rat ventricular trabeculae. J Physiol. 2018 Apr 1;596(7):1153-1166. doi: 10.1113/JP275578.
95. Philip JL, Murphy TM, Schreier DA, Stevens S, Tabima DM, Albrecht M, Frump AL, Hacker TA, Lahm T, Chesler NC. Pulmonary vascular mechanical consequences of ischemic heart failure and implications for right ventricular function. Am J Physiol Heart Circ Physiol. 2019 May 1;316(5):H1167-H1177. doi: 10.1152/ajpheart.00319.2018.
96. Piao L, Fang YH, Parikh KS, Ryan JJ, D'Souza KM, Theccanat T, Toth PT, Pogoriler J, Paul J, Blaxall BC, Akhter SA, Archer SL. GRK2-mediated inhibition of adrenergic and dopaminergic signaling in right ventricular hypertrophy: therapeutic implications in pulmonary hypertension. Circulation. 2012 Dec 11;126(24):2859-69. doi: 10.1161/CIRCULATIONAHA.112.109868.
97. Power AS, Norman R, Jones TLM, Hickey AJ, Ward ML. Mitochondrial function remains impaired in the hypertrophied right ventricle of pulmonary hypertensive rats following short duration metoprolol treatment. PLoS One. 2019 Apr 9;14(4):e0214740. doi: 10.1371/journal.pone.0214740.
98. Pradhan K, Sydykov A, Tian X, Mamazhakypov A, Neupane B, Luitel H, Weissmann N, Seeger W, Grimminger F, Kretschmer A, Stasch JP, Ghofrani HA, Schermuly RT. Soluble guanylate cyclase stimulator riociguat and phosphodiesterase 5 inhibitor sildenafil ameliorate pulmonary hypertension due to left heart disease in mice. Int J Cardiol. 2016 Aug 1;216:85-91. doi: 10.1016/j.ijcard.2016.04.098.
99. Qin T, Kong B, Dai C, Xiao Z, Fang J, Shuai W, Huang H. Protective effects of Dapagliflozin on the vulnerability of ventricular arrhythmia in rats with pulmonary artery hypertension induced by monocrotaline. Bioengineered. 2022 Feb;13(2):2697-2709. doi: 10.1080/21655979.2021.2017652.
100. Radik M, Kmecova Z, Veteskova J, Malikova E, Doka G, Krenek P, Klimas J. Hepatocyte growth factor plays a particular role in progression of overall cardiac damage in experimental pulmonary hypertension. Int J Med Sci. 2019 Jun 2;16(6):854-863. doi: 10.7150/ijms.31690.
101. Ranchoux B, Nadeau V, Bourgeois A, Provencher S, Tremblay É, Omura J, Coté N, Abu-Alhayja'a R, Dumais V, Nachbar RT, Tastet L, Dahou A, Breuils-Bonnet S, Marette A, Pibarot P, Dupuis J, Paulin R, Boucherat O, Archer SL, Bonnet S, Potus F. Metabolic Syndrome Exacerbates Pulmonary Hypertension due to Left Heart Disease. Circ Res. 2019 Aug 2;125(4):449-466. doi: 10.1161/CIRCRESAHA.118.314555.
102. Ravi Y, Selvendiran K, Naidu SK, Meduru S, Citro LA, Bognár B, Khan M, Kálai T, Hideg K, Kuppusamy P, Sai-Sudhakar CB. Pulmonary hypertension secondary to left-heart failure involves peroxynitrite-induced downregulation of PTEN in the lung. Hypertension. 2013 Mar;61(3):593-601. doi: 10.1161/HYPERTENSIONAHA.111.00514.
103. Rawat DK, Alzoubi A, Gupte R, Chettimada S, Watanabe M, Kahn AG, Okada T, McMurtry IF, Gupte SA. Increased reactive oxygen species, metabolic maladaptation, and autophagy contribute to pulmonary arterial hypertension-induced ventricular hypertrophy and diastolic heart failure. Hypertension. 2014 Dec;64(6):1266-74. doi: 10.1161/HYPERTENSIONAHA.114.03261.
104. Sabourin J, Boet A, Rucker-Martin C, Lambert M, Gomez AM, Benitah JP, Perros F, Humbert M, Antigny F. Ca2+ handling remodeling and STIM1L/Orai1/TRPC1/TRPC4 upregulation in monocrotaline-induced right ventricular hypertrophy. J Mol Cell Cardiol. 2018 May;118:208-224. doi: 10.1016/j.yjmcc.2018.04.003.
105. Sakai S, Miyauchi T, Sakurai T, Yamaguchi I, Kobayashi M, Goto K, Sugishita Y. Pulmonary hypertension caused by congestive heart failure is ameliorated by long-term application of an endothelin receptor antagonist. Increased expression of endothelin-1 messenger ribonucleic acid and endothelin-1-like immunoreactivity in the lung in congestive heart failure in rats. J Am Coll Cardiol. 1996 Nov 15;28(6):1580-8. doi: 10.1016/s0735-1097(96)00336-1.
106. Satoh T, Wang L, Espinosa-Diez C, Wang B, Hahn SA, Noda K, Rochon ER, Dent MR, Levine AR, Baust JJ, Wyman S, Wu YL, Triantafyllou GA, Tang Y, Reynolds M, Shiva S, Hilaire CS, Gomez D, Goncharov DA, Goncharova EA, Chan SY, Straub AC, Lai YC, McTiernan CF, Gladwin MT. Metabolic Syndrome Mediates ROS-miR-193b-NFYA-Dependent Downregulation of Soluble Guanylate Cyclase and Contributes to Exercise-Induced Pulmonary Hypertension in Heart Failure With Preserved Ejection Fraction. Circulation. 2021 Aug 24;144(8):615-637. doi: 10.1161/CIRCULATIONAHA.121.053889.
107. Shang L, Yue W, Wang D, Weng X, Hall ME, Xu Y, Hou M, Chen Y. Systolic overload-induced pulmonary inflammation, fibrosis, oxidative stress and heart failure progression through interleukin-1β. J Mol Cell Cardiol. 2020 Sep;146:84-94. doi: 10.1016/j.yjmcc.2020.07.008.
108. Shi ZL, Fang K, Li ZH, Ren DH, Zhang JY, Sun J. EZH2 Inhibition Ameliorates Transverse Aortic Constriction-Induced Pulmonary Arterial Hypertension in Mice. Can Respir J. 2018 Feb 28;2018:9174926. doi: 10.1155/2018/9174926.
109. Shirai M, Beard M, Pearson JT, Sonobe T, Tsuchimochi H, Fujii Y, Gray E, Umetani K, Schwenke DO. Impaired pulmonary blood flow distribution in congestive heart failure assessed using synchrotron radiation microangiography. J Synchrotron Radiat. 2013 May;20(Pt 3):441-8.
110. Sia YT, Lapointe N, Parker TG, Tsoporis JN, Deschepper CF, Calderone A, Pourdjabbar A, Jasmin JF, Sarrazin JF, Liu P, Adam A, Butany J, Rouleau JL. Beneficial effects of long-term use of the antioxidant probucol in heart failure in the rat. Circulation. 2002 May 28;105(21):2549-55. doi: 10.1161/01.cir.0000016721.84535.00.
111. Spyropoulos F, Michael Z, Finander B, Vitali S, Kosmas K, Zymaris P, Kalish BT, Kourembanas S, Christou H. Acetazolamide Improves Right Ventricular Function and Metabolic Gene Dysregulation in Experimental Pulmonary Arterial Hypertension. Front Cardiovasc Med. 2021 Jun 17;8:662870. doi: 10.3389/fcvm.2021.662870
112. Sun F, Lu Z, Zhang Y, Geng S, Xu M, Xu L, Huang Y, Zhuang P, Zhang Y. Stage‑dependent changes of β2‑adrenergic receptor signaling in right ventricular remodeling in monocrotaline‑induced pulmonary arterial hypertension. Int J Mol Med. 2018 May;41(5):2493-2504. doi: 10.3892/ijmm.2018.3449.
113. Tian L, Xiong PY, Alizadeh E, Lima PDA, Potus F, Mewburn J, Martin A, Chen KH, Archer SL. Supra-coronary aortic banding improves right ventricular function in experimental pulmonary arterial hypertension in rats by increasing systolic right coronary artery perfusion. Acta Physiol (Oxf). 2020 Aug;229(4):e13483. doi: 10.1111/apha.13483.
114. Toldo S, Bogaard HJ, Van Tassell BW, Mezzaroma E, Seropian IM, Robati R, Salloum FN, Voelkel NF, Abbate A. Right ventricular dysfunction following acute myocardial infarction in the absence of pulmonary hypertension in the mouse. PLoS One. 2011 Mar 24;6(3):e18102. doi: 10.1371/journal.pone.0018102.
115. Trammell AW, Talati M, Blackwell TR, Fortune NL, Niswender KD, Fessel JP, Newman JH, West JD, Hemnes AR. Pulmonary vascular effect of insulin in a rodent model of pulmonary arterial hypertension. Pulm Circ. 2017 Jul-Sep;7(3):624-634. doi: 10.1086/689908.
116. Tual L, Morel OE, Favret F, Fouillit M, Guernier C, Buvry A, Germain L, Dhonneur G, Bernaudin JF, Richalet JP. Carvedilol inhibits right ventricular hypertrophy induced by chronic hypobaric hypoxia. Pflugers Arch. 2006 Jul;452(4):371-9. doi: 10.1007/s00424-006-0058-5.
117. Umar S, Lee JH, de Lange E, Iorga A, Partow-Navid R, Bapat A, van der Laarse A, Saggar R, Saggar R, Ypey DL, Karagueuzian HS, Eghbali M. Spontaneous ventricular fibrillation in right ventricular failure secondary to chronic pulmonary hypertension. Circ Arrhythm Electrophysiol. 2012 Feb;5(1):181-90. doi: 10.1161/CIRCEP.111.967265.
118. Umar S, Ruffenach G, Moazeni S, Vaillancourt M, Hong J, Cunningham C, Cao N, Navab S, Sarji S, Li M, Lee L, Fishbein G, Ardehali A, Navab M, Reddy ST, Eghbali M. Involvement of Low-Density Lipoprotein Receptor in the Pathogenesis of Pulmonary Hypertension. J Am Heart Assoc. 2020 Jan 21;9(2):e012063. doi: 10.1161/JAHA.119.012063.
119. Usui S, Yao A, Hatano M, Kohmoto O, Takahashi T, Nagai R, Kinugawa K. Upregulated neurohumoral factors are associated with left ventricular remodeling and poor prognosis in rats with monocrotaline-induced pulmonary arterial hypertension. Circ J. 2006 Sep;70(9):1208-15. doi: 10.1253/circj.70.1208.
120. Vázquez-Garza E, Bernal-Ramírez J, Jerjes-Sánchez C, Lozano O, Acuña-Morín E, Vanoye-Tamez M, Ramos-González MR, Chapoy-Villanueva H, Pérez-Plata L, Sánchez-Trujillo L, Torre-Amione G, Ramírez-Rivera A, García-Rivas G. Resveratrol Prevents Right Ventricle Remodeling and Dysfunction in Monocrotaline-Induced Pulmonary Arterial Hypertension with a Limited Improvement in the Lung Vasculature. Oxid Med Cell Longev. 2020 Feb 3;2020:1841527. doi: 10.1155/2020/1841527.
121. Wang L, Halliday G, Huot JR, Satoh T, Baust JJ, Fisher A, Cook T, Hu J, Avolio T, Goncharov DA, Bai Y, Vanderpool RR, Considine RV, Bonetto A, Tan J, Bachman TN, Sebastiani A, McTiernan CF, Mora AL, Machado RF, Goncharova EA, Gladwin MT, Lai YC. Treatment With Treprostinil and Metformin Normalizes Hyperglycemia and Improves Cardiac Function in Pulmonary Hypertension Associated With Heart Failure With Preserved Ejection Fraction. Arterioscler Thromb Vasc Biol. 2020 Jun;40(6):1543-1558. doi: 10.1161/ATVBAHA.119.313883.
122. Wang Q, Guo YZ, Zhang YT, Xue JJ, Chen ZC, Cheng SY, Ou MD, Cheng KL, Zeng WJ. The Effects and Mechanism of Atorvastatin on Pulmonary Hypertension Due to Left Heart Disease. PLoS One. 2016 Jul 7;11(7):e0157171. doi: 10.1371/journal.pone.0157171.
123. Wang X, Xu Q, Li T, Rong Y, Hong W, Huang Y, Guo X. Intratracheal administration of isosorbide dinitrate improves pulmonary artery pressure and ventricular remodeling in a rat model of heart failure following myocardial infarction. Exp Ther Med. 2017 Aug;14(2):1399-1408. doi: 10.3892/etm.2017.4707.
124. Wang Y, Tian W, Xiu C, Yan M, Wang S, Mei Y. Urantide improves the structure and function of right ventricle as determined by echocardiography in monocrotaline-induced pulmonary hypertension rat model. Clin Rheumatol. 2019 Jan;38(1):29-35. doi: 10.1007/s10067-018-3978-5.
125. Wittmer VL, Waichert ÉJ, Gava PL, Pereira FE, Guimarães MC, de Figueiredo SG, Mauad H. Effects of captopril on cardiovascular reflexes and respiratory mechanisms in rats submitted to monocrotaline-induced pulmonary arterial hypertension. Pulm Pharmacol Ther. 2015 Feb;30:57-65. doi: 10.1016/j.pupt.2014.11.001.
126. Wu J, Liu T, Shi S, Fan Z, Hiram R, Xiong F, Cui B, Su X, Chang R, Zhang W, Yan M, Tang Y, Huang H, Wu G, Huang C. Dapagliflozin reduces the vulnerability of rats with pulmonary arterial hypertension-induced right heart failure to ventricular arrhythmia by restoring calcium handling. Cardiovasc Diabetol. 2022 Sep 28;21(1):197. doi: 10.1186/s12933-022-01614-5.
127. Xiong PY, Baba S, Nishioka N, Fujimoto Y, Archer SL, Minamisawa S. Left Atrial Stenosis Induced Pulmonary Venous Arterialization and Group 2 Pulmonary Hypertension in Rat. J Vis Exp. 2018 Nov 18;(141). doi: 10.3791/58787.
128. Xiong PY, Motamed M, Chen KH, Dasgupta A, Potus F, Tian L, Martin A, Mewburn J, Jones O, Thébaud A, Archer SL. Inhibiting pyruvate kinase muscle isoform 2 regresses group 2 pulmonary hypertension induced by supra-coronary aortic banding. Acta Physiol (Oxf). 2022 Feb;234(2):e13764. doi: 10.1111/apha.13764.
129. Yin J, Kukucka M, Hoffmann J, Sterner-Kock A, Burhenne J, Haefeli WE, Kuppe H, Kuebler WM. Sildenafil preserves lung endothelial function and prevents pulmonary vascular remodeling in a rat model of diastolic heart failure. Circ Heart Fail. 2011 Mar;4(2):198-206. doi: 10.1161/CIRCHEARTFAILURE.110.957050.
130. Yin N, Kaestle S, Yin J, Hentschel T, Pries AR, Kuppe H, Kuebler WM. Inhaled nitric oxide versus aerosolized iloprost for the treatment of pulmonary hypertension with left heart disease. Crit Care Med. 2009 Mar;37(3):980-6. doi: 10.1097/CCM.0b013e3181962ce6.
131. Yue W, Tong L, Liu X, Weng X, Chen X, Wang D, Dudley SC, Weir EK, Ding W, Lu Z, Xu Y, Chen Y. Short term Pm2.5 exposure caused a robust lung inflammation, vascular remodeling, and exacerbated transition from left ventricular failure to right ventricular hypertrophy. Redox Biol. 2019 Apr;22:101161. doi: 10.1016/j.redox.2019.101161
132. Zaky A, Zafar I, Masjoan-Juncos JX, Husain M, Mariappan N, Morgan CJ, Hamid T, Frölich MA, Ahmad S, Ahmad A. Echocardiographic, Biochemical, and Electrocardiographic Correlates Associated With Progressive Pulmonary Arterial Hypertension. Front Cardiovasc Med. 2021 Jul 20;8:705666. doi: 10.3389/fcvm.2021.705666.
133. Zhang H, Huang W, Liu H, Zheng Y, Liao L. Mechanical stretching of pulmonary vein stimulates matrix metalloproteinase-9 and transforming growth factor-β1 through stretch-activated channel/MAPK pathways in pulmonary hypertension due to left heart disease model rats. PLoS One. 2020 Sep 3;15(9):e0235824. doi: 10.1371/journal.pone.0235824.
134. Zhang YT, Xue JJ, Wang Q, Cheng SY, Chen ZC, Li HY, Shan JJ, Cheng KL, Zeng WJ. Dehydroepiandrosterone attenuates pulmonary artery and right ventricular remodeling in a rat model of pulmonary hypertension due to left heart failure. Life Sci. 2019 Feb 15;219:82-89. doi: 10.1016/j.lfs.2018.12.056.
135. Zhuang R, Wu J, Lin F, Han L, Liang X, Meng Q, Jiang Y, Wang Z, Yue A, Gu Y, Fan H, Zhou X, Liu Z. Fasudil preserves lung endothelial function and reduces pulmonary vascular remodeling in a rat model of end‑stage pulmonary hypertension with left heart disease. Int J Mol Med. 2018 Sep;42(3):1341-1352. doi: 10.3892/ijmm.2018.3728.
